# The relationship between coffee-related factors and cortical and hippocampal structure: a triangulation of evidence approach and Mendelian randomization research

**DOI:** 10.3389/fnut.2024.1351067

**Published:** 2024-05-21

**Authors:** Zining Luo, Lijun Xiong, Xinyu Xu, Meng Sun, Yingfei Mu, Hongjie Chen, Zhenglong Liu, Zhiyong Luo, Jianli Wang, Ying Liu

**Affiliations:** ^1^Department of Preclinical Medicine and Forensic Medicine, North Sichuan Medical College, Nanchong, China; ^2^Department of Stomatology, North Sichuan Medical College, Nanchong, China; ^3^Department of Anesthesiology, North Sichuan Medical College, Nanchong, China; ^4^Department of Clinical Medicine, North Sichuan Medical College, Nanchong, China; ^5^College of Earth Sciences, Chengdu University of Technology, Chengdu, China; ^6^Department of Encephalopathy, Pidu District Traditional Chinese Medicine Hospital, Chengdu, China; ^7^Department of Stomatology, Affiliated Hospital of North Sichuan Medical College, Nanchong, China

**Keywords:** coffee, caffeine, metabolites, cerebral cortex, hippocampal, Mendelian randomization

## Abstract

**Objective:**

Existing studies have reported sustained changes in the cortical structure of rats due to coffee-related factors, which are speculated to occur in the human body. However, there is a lack of research on this topic. Additionally, previous observational studies have found the impact of diseases on cortical structure and the potential therapeutic effects of coffee on these diseases. Our aim was to study the causal effects of coffee-related factors on the human brain using SNPs (single nucleotide polymorphisms). We will connect these discovered causal effects to the impact of diseases on the brain. Through triangulating evidence, we will reveal the potential active areas of coffee in preventing diseases.

**Methods:**

We utilized GWAS data from multiple cohorts and their databases, selecting instrumental variables for genetic prediction of coffee intake and plasma levels of caffeine and its direct metabolites. We applied these instrumental variables to individual data on cortical thickness and surface area, as well as hippocampal volume, from the ENIGMA and CHARGE consortium for Mendelian randomization analysis (MR). Triangular evidence was obtained by integrating existing evidence through a specified retrieval strategy, calculating the overlap between coffee's effects on brain regions and disease-related brain regions to identify potential regions of action.

**Results:**

The MR analysis yielded 93 positive results for 9 exposures, among which theobromine, a metabolite in the caffeine pathway, was found to be associated with increased hippocampal volume. For cortical structure, theobromine in the caffeine pathway was associated with a decrease in total surface area, while theobromine and caffeine in the pathway were associated with an increase in total thickness. The overlap rate of triangular evidence showed no difference in both overall and subgroup analyses, indicating a high overlap between the effects of coffee on brain regions and disease.

**Conclusions:**

From predicted outcomes from causal effects, coffee intake-related factors may have lasting effects on cortical structure. Additionally, theobromine and theophylline have the greatest impact on certain brain gyri, rather than caffeine. Triangulation evidence indicates that disease and coffee intake-related factors act on the same cortical regions, suggesting the presence of potential shared or antagonistic pathways.

## 1 Background

Coffee is currently one of the most widely consumed beverages around the world, and the demand is significantly increasing. According to statistics from the United States Department of Agriculture (USDA) (1), from 2013 to 2022, global coffee production increased by approximately 9.31%. Additionally, there was a 15.98% increase in global coffee imports. Therefore, the impact of coffee on the human body has long been a topic of great interest. Research on the effects of coffee, a beverage that provides energy and stimulation (2), on the brain has been conducted (3). In 1975, Aeschbacher et al. conducted a study on rats and proposed that coffee shortens the duration of drug effects by altering brain structure through its interaction with the brain (4). With the advancement of brain structure classification and the development of MRI technology, research on the specific morphology of coffee and the brain's cortical regions, such as thickness (5), has gradually increased. In addition to the brain, the debate about the pros and cons of coffee for human health has been ongoing for a long time (6). In clinical studies with positive outcomes, coffee has been found to have various benefits, such as promoting physical activity (7), lowering the risk of depression (8), reducing the risk of Parkinson's disease (9), and lowering the risk of type 2 diabetes (10). However, Burke et al. also reported that excessive coffee consumption disrupts the body's circadian rhythm (11).

Previous studies have shown that coffee has lasting effects on the brain. In animal experiments, regular intake of caffeine has been shown to improve the signal-to-noise ratio in information encoding processes (12). In clinical studies, changes in the morphological structure of the cerebral cortex have also been observed in relation to coffee intake (13). In Mendelian randomization studies (MR), it has been found that higher coffee consumption is associated with a reduction in brain gray matter volume (14). However, previous studies also have significant limitations. The research design of animal experiments cannot be directly applied to the human body (15). The small sample size in clinical studies leads to insufficient statistical robustness (16). The long-term effects of coffee on the brain, the effects of diseases on the brain, and the potential shared pathways or regions involved have not been elucidated. Current MR studies have not conducted causal analyses on the specific processes of coffee intake and the morphology of various brain regions. At the same time, prospective cohort studies that have shown a certain causal effect have not been conducted, leading to uncertainty regarding the causal relationship between coffee intake and changes in brain morphology.

MR utilizes genetic variants as instrumental variables, aiding in overcoming confounding biases in clinical research (17, 18). This approach establishes causal relationships between risk factor exposures and disease outcomes. Our study employed publicly available large-scale genome-wide association study (GWAS) data to conduct multiple two-sample MR analyses. These analyses aimed to investigate the impact of coffee-related factors on cerebral cortical structures. Additionally, we conducted a triangulation analysis to elucidate potential relationships among coffee-related factors, cerebral cortex structures, and various diseases.

In summary, our study aimed to address the limitations of previous animal experiments and clinical research while uncovering the causal effects of coffee on the morphology of the cerebral cortex and hippocampal regions. By exploring the potential relationships among coffee-related factors, cerebral cortex structures, and various diseases, we offer insights and potential directions for future research into the collective influences of coffee on brain structure and various disease mechanisms.

## 2 Methods

### 2.1 Study protocol

The literature review followed the preferred reporting items for systematic reviews and meta-analyses (PRISMA (19)) guidelines. Our protocol was registered on the PROSPERO network platform on October 1, 2023, with the registration number CRD42023468538. Additionally, the description of the MR analysis section responds to the call of the Journal of Clinical Endocrinology & Metabolism (JECM) and was designed and implemented using the STROBE-MR checklist (20).

### 2.2 Data sources for coffee intake, caffeine, theobromine, theophylline, and paraxanthine

**Figure 1:**
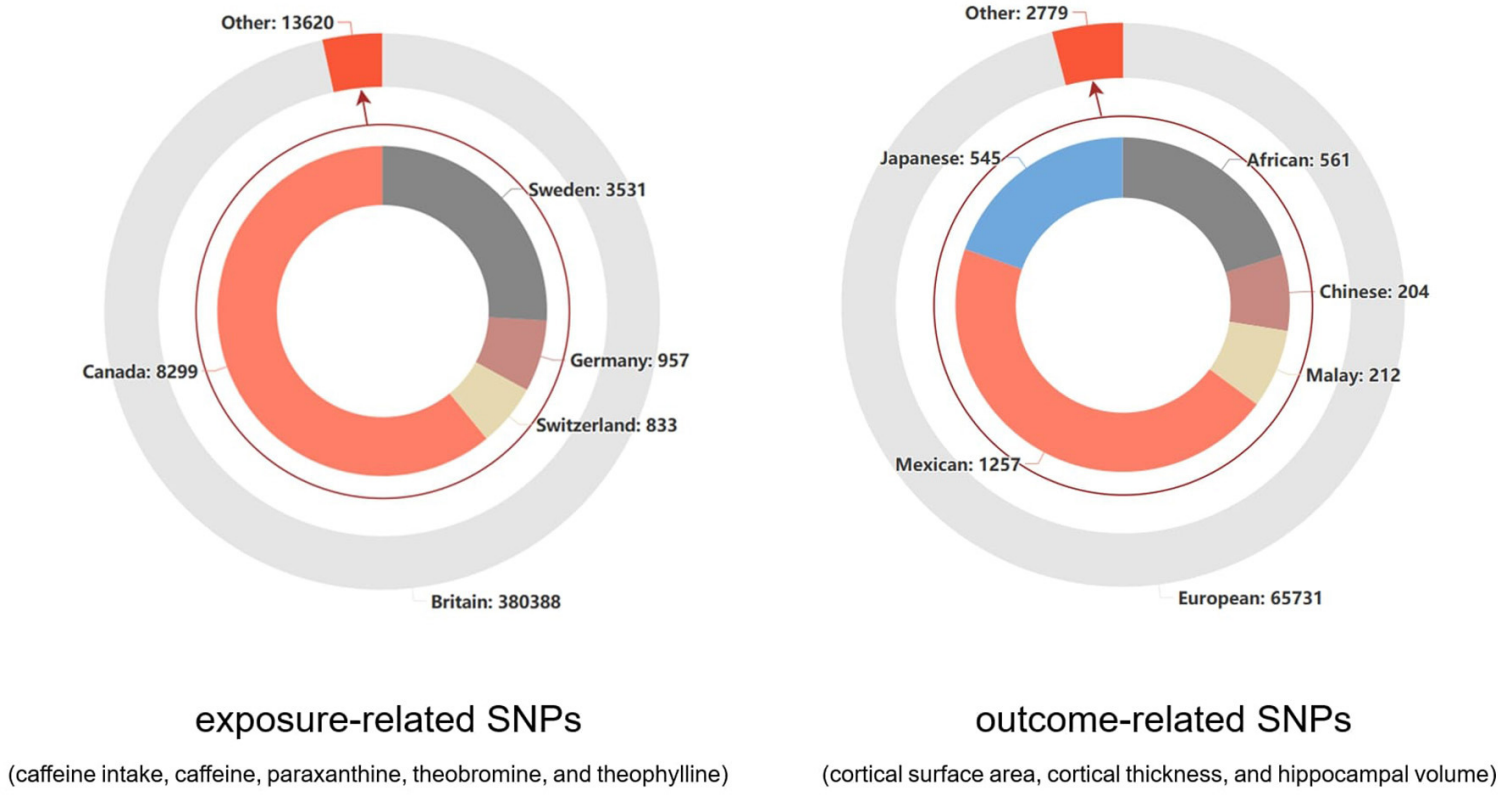
The distribution of study populations corresponding to the SNPs related to outcomes and exposure factors.

For the exposure-related process of coffee intake, the data on caffeine and the other direct metabolite SNPs on their metabolic pathways were sourced from the UK Biobank database (*n* = 375,833) (21) and a meta-analysis of GWAS summary statistics from six European ancestry populations (*n* = 9,876) (22). Additionally, we studied the inherent presence of caffeine, theobromine, theophylline, and xanthine in plasma, with their related SNPs sourced from individuals of European ancestry in the Canadian Longitudinal Study on Ageing (CLSA) cohort (*n* = 8,299) (23). All the information from these cohorts involved in this study is listed in Supplementary Table S1. Regarding the standard unit of exposure, coffee, the SNP-coffee association was described in detail by Yuan et al. (24), where the effect size was increased by 50% for each standard unit increase in coffee consumption (e.g., from 1 cup to 1.5 cups). In the studies conducted by Cornelis (22) and Chen et al. (23), caffeine and its metabolites under two distinct conditions were thoroughly studied. All the data were standardized to a mean of zero and a standard deviation of one. In terms of the regional distribution of the SNP sources from the various cohorts, the total number of individuals was 394,008, with a predominant representation of European populations (97.9%). The specific distribution is shown in Figure 1.

### 2.3 Data sources for the cortical surface area, cortical thickness, and hippocampal volume

For the outcome-related process, the GWAS data related to cortical brain structure were sourced from the ENIGMA consortium (25). They conducted the research using multiple individual brain magnetic resonance imaging data (*n* = 51,665) and analyzed the surface area and average thickness of the entire cortex, as well as 34 functionally specialized regions. The GWAS data related to hippocampal volume were sourced from another collaboration between the ENIGMA consortium and the CHARGE consortium (26). This GWAS analysis was based on MRI scans and whole-genome genotyping data, with participants from 65 sites (*n* = 33,536). The cohort information for these two studies is provided in Supplementary Tables S2, S3. The 34 regions of the cerebral cortex were defined based on the Desikan-Killiany atlas, and the regions between the two hemispheres were averaged (27). For the hippocampus, the FSLFIRST algorithm (28) was used to estimate hippocampal volume. In terms of the regional distribution of SNP sources from different cohorts, the European population accounted for an overwhelming majority (95.9%), as shown in Figure 1.

### 2.4 Selection of genetic instruments

We identified two main exposure groups: (1) those involving coffee intake and (2) those involving caffeine and its direct metabolites. For coffee intake, we used a set of genetic tools, while for caffeine and its direct metabolites, we utilized two sets of genetic tools, encompassing their presence both in the plasma and within metabolic pathways.

To obtain genetic tools with sufficient significance and an adequate number of samples for analysis, we conducted testing and preliminary screening of genetic tools using different *P*-values (Supplementary Table S10). Additionally, we required a linkage disequilibrium (LD) r2 value of < 0.001 and a distance of < 1 MB from the index variant.

In the initial phase of our analysis, we excluded SNPs that had *P*-value thresholds corresponding to the brain's cerebral cortex or hippocampus volume. Additionally, we utilized PhenoScanner (http://www.phenoscanner.medschl.cam.ac.uk/) (29, 30) to identify SNPs potentially associated with confounding factors. Following discussions with Jianli Wang and a preliminary analysis, a subset of these SNPs was further excluded.

We considered the independence of the samples using two principles (31): (1) reading the original literature to determine there was no overlap between the exposed and outcome cohorts and (2) calculating the maximum estimated overlap rate. Independence was determined based on compliance with Principle 1, with thresholds for maximum estimated overlap set at 25% and 10%.

Finally, for the potential SNPs that may not exist in the outcome, proxy SNPs were used for substitution analysis (32).

### 2.5 Mendelian randomization analyses

After the selection of SNPs was completed, MR analysis was conducted based on two different scenarios.

**Figure 2:**
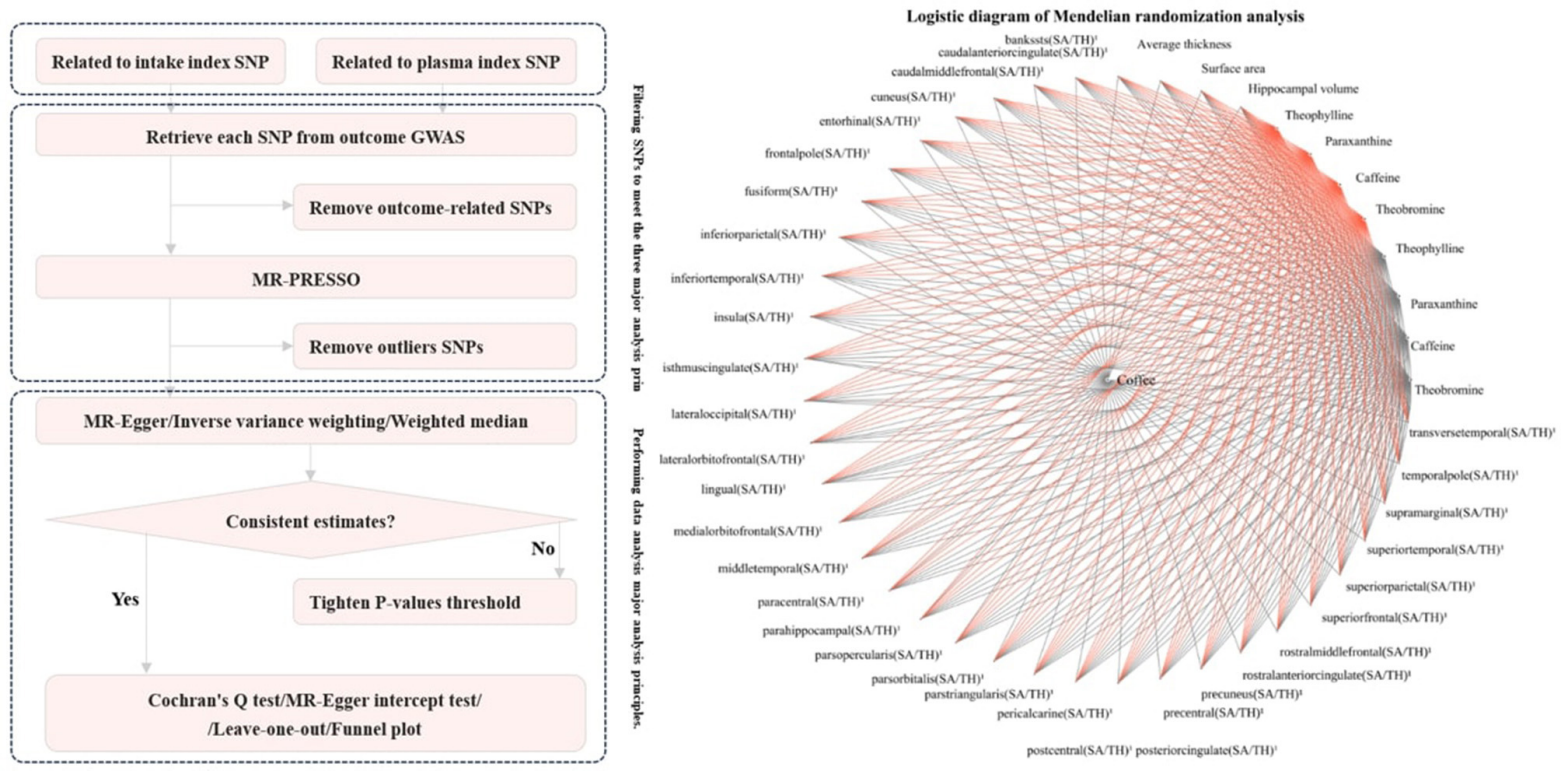
Flowchart of MR analysis for exposure factors with more than 2 SNPs.

For exposure factors with more than two SNPs, three different MR methods were used under the random effects model: inverse variance weighting (IVW), MR-Egger, and weighted median. IVW was used as the primary result, while MR-Egger and the weighted median were used to estimate the IVW result. MR-Egger was used to assess pleiotropy by considering the presence of an intercept term in the regression (33). The weighted median allows for the inclusion of invalid instrumental variables in the analysis, provided that most instruments are valid. Hence, their results can improve the reference of IVW results. After adjusting the threshold for the agreed-upon *p*-value, sensitivity analyses were performed on each important analysis result using MR-Egger, IVW, Cochran's Q test, and leave-one-out analysis. MR-Egger and IVW were used for heterogeneity detection, with IVW heterogeneity detection being the primary result, and MR-Egger was used for supplementary interpretation. Although the previous MR-PRESSO studies had already screened and discarded results with high heterogeneity, leave-one-out analysis can further identify specific sources of heterogeneity within an acceptable range. Finally, a funnel plot was used to visualize heterogeneity. The overall workflow of all the analyses is presented in Figure 2.

For exposure factors with SNP numbers ≤ 2, the IVW method based on weighted least squares provides a more efficient estimate. However, it is not suitable for analyzing outcomes with a single SNP. The Wald ratio, on the other hand, can be used for analyzing outcomes with a single SNP. It is based on comparing ratios and assumes a linear effect of the genetic variant. Although it is more prone to bias, in this case, we chose to analyze the two SNPs using the IVW method based on a random effects model and to analyze the single SNP using the Wald ratio (34).

After determining significant outcomes based on *p-*values, when the IVW, MR-Egger, and weighted median methods had inconsistent directions, this portion of the results was discarded. When the directions of all three methods were consistent, we consider it to have a significant effect. However, when it was not possible to perform MR-Egger or weighted median analysis due to the number of SNPs or when only Wald ratio analysis could be conducted with potentially larger errors, we considered it a nominally significant effect. In such cases, we used the Wald ratio or IVW estimate as the result.

### 2.6 Triangular evidence on the impact on the cerebral cortex

**Figure 3:**
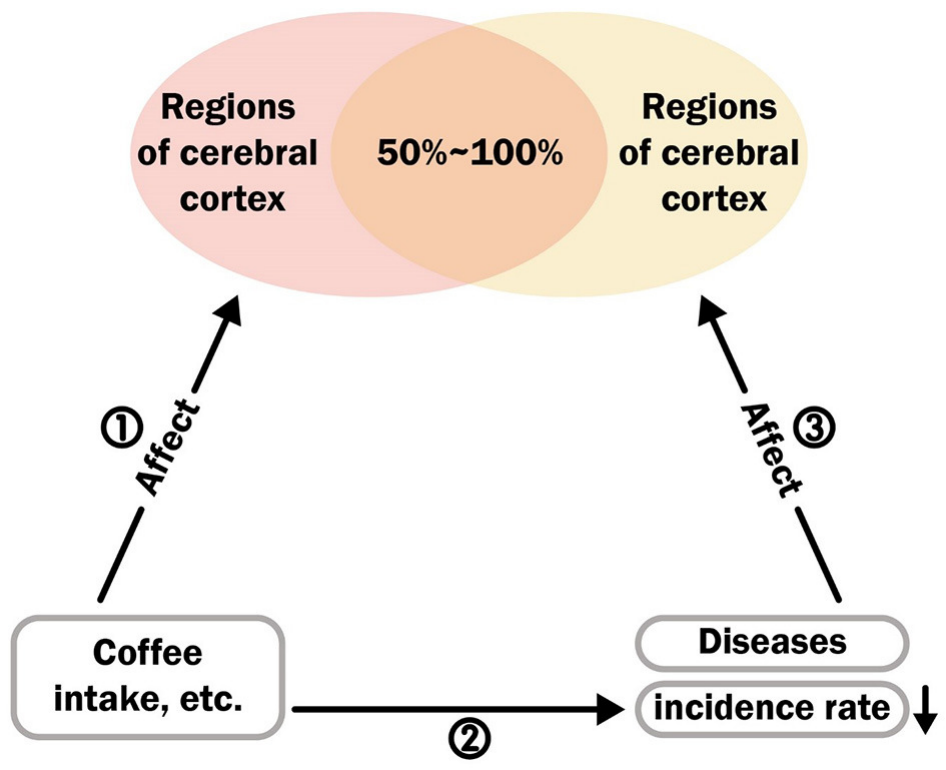
The logical relationship between disease, cerebral cortex, and coffee intake.

Existing research has revealed the impact of diseases on the cerebral cortex. With the help of MR analysis, we can discover the causal effects of coffee, caffeine, and their metabolites on the cerebral cortex. Furthermore, some observational and corresponding MR studies have identified the positive effects of coffee intake on these diseases (this logic is presented in Figure 3). We have collected epidemiological evidence and utilized the Newcastle-Ottawa Scale (NOS) (35) and the Agency for Healthcare Research and Quality (AHRQ) Scale (36) for quality assessment. For the NOS, it employs a star system ranging from 0 to 9 to evaluate participant selection and exposure measurement. A higher score reflects superior study quality, with scores ranging from 7 to 9 indicating high quality. Regarding the AHRQ, it uses a system of 11 inherent questions to evaluate studies. Additionally, we calculated the number of regions in the cerebral cortex affected by diseases and attempted to calculate the overlap rate with the regions affected by coffee, caffeine, and their metabolites. By combining the overlap rate with epidemiological evidence, a higher overlap rate indicates that diseases and coffee act on the same regions of the cerebral cortex through similar mechanisms. The results of this analysis can narrow the selection range for disease pathways or targets. By retrospectively verifying the specific regions of the brain that are positively affected by coffee and other related substances in relation to a particular disease, we can provide further evidence for precise pathway selection. We selected four common diseases (gait disorders, major depressive disorder, Parkinson's disease and type 2 diabetes) and one specific behavior (suicide) as specific outcomes to study the potential similarities and antagonistic effects between the mechanisms of disease on the brain and the effects of coffee or other factors on the brain.

### 2.7 Search approach

To obtain information on the impact of these diseases on cortical morphology and the effects of factors such as coffee intake on these diseases, we searched the PubMed database up to November 1, 2023. We used Medical Subject Headings (MeSH) and relevant keywords. The search terms included “gait disorders,” “cortical morphology,” “type 2 diabetes,” etc. The complete search strategies for each item can be found in Supplementary Table S4.

### 2.8 Eligibility criteria

The inclusion criteria were divided into two categories. First, for studies on coffee and disease, (1) the exposure factor had to be related to coffee intake, such as the number of cups/day or high coffee intake population. (2) Outcome variables related to the five major diseases or factors were considered. (3) Only observational studies were included. Second, for studies on coffee and cortical brain regions, since the results obtained in MR analysis were averaged across hemispheres, the following criteria were used: (1) The exposure factor had to be related to the five major diseases or factors considered. (2) Clinical contrast studies based on MRI. (3) Outcome variables only considered the average surface area or thickness of the displayed results, or the surface area or thickness of both the left and right hemispheres were included in the displayed results.

### 2.9 Article confirmation and information collection

The article's confirmation and information collection processes were carried out by the first reviewer (LZN) and the second reviewer (XLJ). After completion, cross-validation and consultation were conducted with a third (CHJ). A predesigned data extraction form was used, and the following data were extracted: outcome factors, first author, publication year, cohort source, outcome definition, corresponding MR studies, and age. Any potential differences were resolved through discussion with the third reviewer (CHJ) to reach a consensus.

### 2.10 Statistical analysis

The main analyses were conducted using the software packages TwoSampleMR (version 0.5.7) and MRPRESSO (version 1.0) in R (version 4.3.1). Prior to the analysis, the removal of confounding SNPs was performed using PhenoScanner (http://www.phenoscanner.medschl.cam.ac.uk/) (29, 30) and our custom software PhenoScanner-Results-Processor (https://github.com/Ruosining/PhenoScanner-Results-Processor), which was created using vb.net (.NET Framework 4.8). At the global level of testing (including hippocampal volume and cortical regions), a significant two-sided *p* value was set at 0.05. At the regional level of testing (including 34 cortical regions only), given 1224 MR estimates, the Bonferroni-corrected *p* value was set at 0.05/1224 (4.08e-5). Additionally, we retained *p* < 0.05 as nominally significant (37).

## 3 Results

### 3.1 Mendelian randomization results

After phenotype screening using the PhenoScanner platform, reading the relevant literature for each phenotype, and triple confirmation by Jianli Wang, we excluded SNPs associated with certain potential confounders, as detailed in Supplementary Table S11.

**Figure 4:**
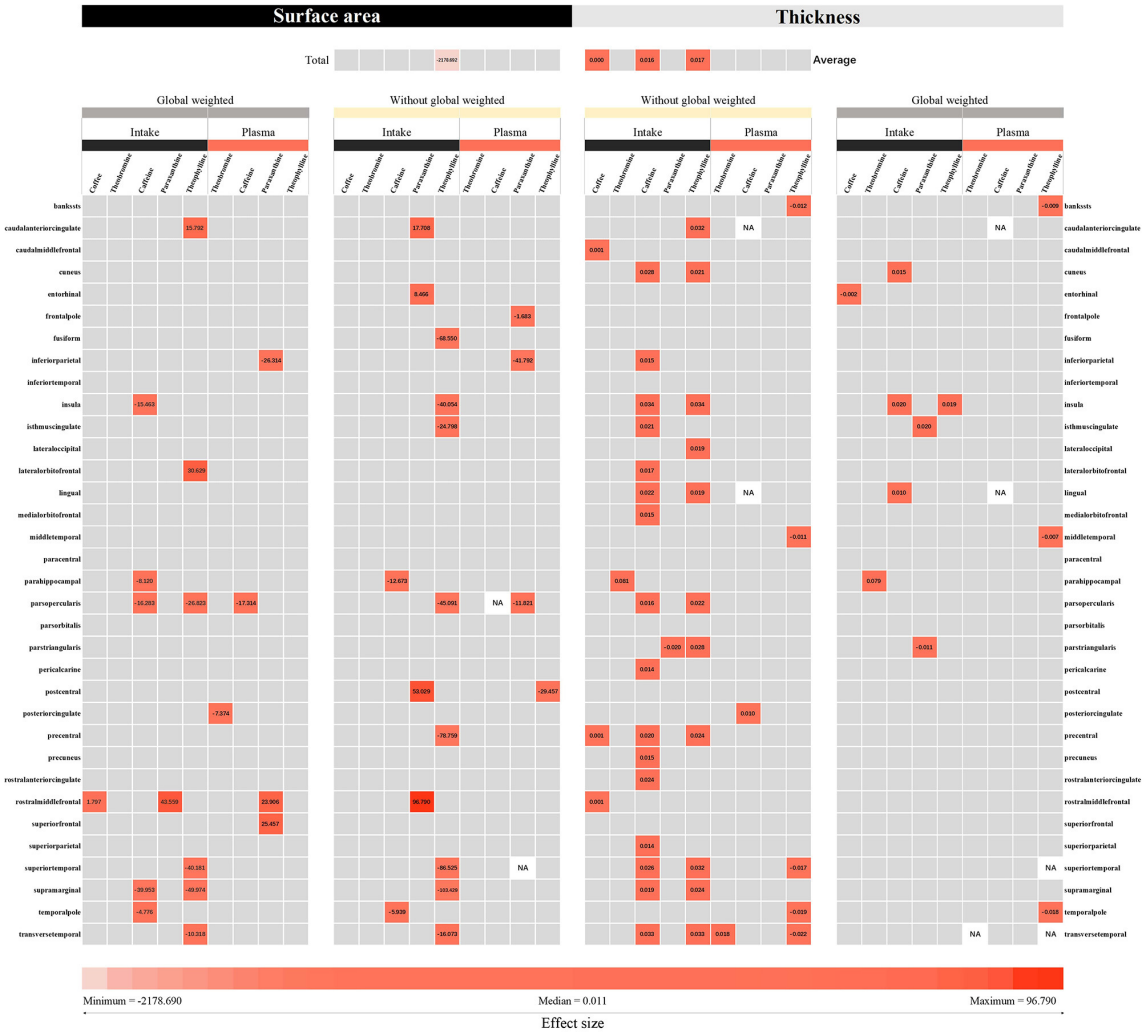
The IVW and Wald ratio methods were used to estimate the intake of coffee and its direct metabolic products, as well as the plasma levels of caffeine, theobromine, theophylline, and paraxanthine in the plasma metabolic pathway. The effects of these compounds on cortical thickness and surface area were measured using MRI. The effect sizes (beta) representing the IVW or Wald ratio method were derived based on the intensity of color blocks. The numerical values displayed in the color blocks represent the corresponding effect sizes. The gray blocks represent results that are not statistically significant. According to our defined criteria, *p* values below 0.05 were considered nominally significant, while those below the threshold of 4.08e-5 were considered significant.

After screening, a total of 63 SNP indicators were included for genetic prediction of coffee intake and other exposures. These SNPs are presented in Supplementary Table S5. Overall, the F-statistics for these genetic instruments were all >20, indicating that the instruments were strong (38). We conducted a comprehensive MR analysis of 34 brain regions and the hippocampus, focusing on nine different exposures. A total of 1251 analyses were performed. The results, primarily based on the IVW method, yielded 93 positive findings (as shown in Figure 4).

Among the results with a *P*-value < 0.05 for the IVW or Wald ratio, 30.1% were found to be significant. In the analysis with hippocampal volume as the outcome variable, theophylline, a pathway metabolite of caffeine in plasma, was found to increase hippocampal volume (β = 0.169 mm^3^, 95% CI: 0.016 mm^3^ to 0.323 mm^3^, *P* = 0.031). According to our analysis with cortical surface area as the outcome variable, theophylline in plasma, a metabolite in the caffeine metabolic pathway, was found to be associated with a decrease in cortical surface area overall (β = −2,178.690 mm^2^, 95% CI: −4,265.520 mm^2^ to −91.867 mm^2^, *P* = 0.041). Theophylline (β = 0.017 mm, 95% CI: 0.003 mm to 0.031 mm, *P* = 0.018) and caffeine (β = 0.016 mm, 95% CI: 0.007 mm to 0.025 mm, *P* = 0.000) in plasma, both of which are metabolites in the caffeine pathway, were found to be associated with an increase in cortical thickness. An increase in cortical thickness was also associated with the consumption of 50% more coffee (β = 0.0004 mm, 95% CI: 0.000 mm to 0.001 mm, *P* = 0.005). No causal relationships were found between other exposures and the cortical surface area, total hippocampal volume, or hippocampal volume. Since the IVW method was pre-specified as the primary approach with a random-effects linear IVW model, the observed heterogeneity with a Cochran Q *p-*value < 0.05 was accepted. Sensitivity analyses for the results with enough SNPs did not reveal any multiplicative effects, and no outliers were found in the MR-PRESSO analysis, leave-one-out analysis, or funnel plots (Supplementary Figure S1).

According to the analysis of 34 functional regions, without global weighting, theophylline in the caffeine pathway, had the greatest impact on the surface area of the supramarginal gyrus (β = −103.429 mm^2^, 95% CI: −173.819 mm^2^ to −33.039 mm^2^, *P* = 0.004). With global weighting, theophylline in the caffeine pathway had the greatest impact on the surface area of the supramarginal gyrus (β = −49.974 mm^2^, 95% CI: −97.443 mm^2^ to −2.504 mm^2^, *P* = 0.039). The caffeine metabolite theobromine had the greatest impact on the thickness of the parahippocampal gyrus without global weighting (β = 0.081 mm, 95% CI: 0.011 mm to 0.150 mm, *P* = 0.023). With respect to global weight, theobromine in the caffeine pathway had the greatest impact on the thickness of the parahippocampal gyrus (β = 0.079 mm, 95% CI: 0.012 mm to 0.146 mm, *P* = 0.020). Other brain regions with significant or nominally significant findings are displayed in Supplementary Figure S2.

The results of other brain regions with significant or nominally significant findings are displayed in Supplementary Figure S2. Sensitivity analyses, including the Cochran's Q test, MR-Egger test, leave-one-out analysis, and funnel plots, were conducted for a sufficiently large number of SNP exposures, and no evidence of pleiotropy or significant bias was found. The observed heterogeneity was accepted, and no significant bias was detected in the leave-one-out analysis for the individual SNPs (Supplementary Figure S3).

### 3.2 Triangular evidence

To reflect the potential shared or antagonistic mechanisms between coffee intake and disease factors, which are ultimately manifested in the concentration of brain regions through the cerebral cortex, we summarized the evidence from three aspects in Figure 3 and illustrated this through the overlapping rates of brain regions influenced by the pathways ① and ③. First, for pathway ①, in the MR analysis results, we collected and counted the effects of coffee intake, caffeine and its direct metabolites on the brain regions influenced by theobromine, theophylline, and xanthine. The final results are shown in Supplementary Table S9.

For pathway ②, the retrieval process and selection diagram are listed in Supplementary Figure S4. Five types of observational studies were included to examine the impact of coffee intake on disease incidence. Most of these articles confirm that coffee reduces the risk of potential diseases. Detailed information on these studies is presented in Supplementary Table S6. Excluding some cross-sectional studies that did not show a significant causal relationship, the other studies provided direct evidence for the existence of pathway ②.

For pathway ③, a total of 7 clinical comparative studies involving 14,985 individuals were included (39–45). The age range of the participants was from 9 to 80 years, and their detailed characteristics are presented in Supplementary Table S7. Since MRI-based clinical comparative studies cannot reflect the causal relationship between brain regions and diseases, they provide evidence for the potential existence of pathway ③.

The final quality assessment for pathway ② and ③ revealed that the average score on the NOS was 7 (ranging from 6 to 8), indicating good research quality. For additional detailed results, please refer to Supplementary Table S12.

### 3.3 Overlap rate of brain regions

In each study included in pathway ③, shown in Figure 3, the impact of disease on the brain regions of the left and right hemispheres was collected (Supplementary Table S8). Finally, the brain regions included in this case, which had an impact on disease in both the left and right hemispheres, were combined with the overall brain regions affected by coffee, caffeine metabolites, theobromine, theophylline, and xanthine (without subgroup adjustment) and the brain regions that underwent subgroup adjustment (including global weighted and non-global weighted) was used to calculate the intersection. The number of common brain regions was divided by the total number of the overall brain regions (without subgroup adjustment) and the number of brain regions that underwent subgroup adjustment (including global weighted and non-global weighted) were used to calculate the overlapping rate of brain regions. The final results are presented in Supplementary Table S9.

Due to the limitations of the current research, clinical control studies have only investigated the correlation between diseases such as gait disorders and the thickness of a few brain regions. The study on suicidal ideation only examined the correlation with brain region surface area. Regardless of whether subgroup analysis was conducted or not and whether global weighting was performed or not, these diseases all demonstrated a relatively high brain region overlap rate (40%−100%).

## 4 Discussion

As of November 1, 2023, this is the first comprehensive large-scale MR analysis in which we investigated the causal effects of coffee intake, caffeine, and their direct metabolites on brain cortical structure and hippocampal volume. At the same time, this is also the first attempt in neuroscience to use evidence of overlap rates with triangular logic relationships to speculate on common or antagonistic pathways of coffee intake and related factors that may affect certain diseases. Our research findings indicate that coffee, caffeine, and their direct metabolites or their inherent presence in plasma can have an impact on the brain cortex and may have direct or indirect causal relationships. This finding supports the theory proposed by previous scholars that coffee intake may directly affect the brain (46). Furthermore, interpreting the overlap and rate of overlap between various diseases and cortical coffee intake reveals indicate potential targets or pathways for treating or delaying diseases. This significantly narrows the scope of the recently proposed brain-gut axis in the specific application of disease-coffee interactions. Contrary to popular belief (47), we observed that caffeine is not the major factor influencing the size of the brain cortex on the predicted metabolic pathways. In fact, at least at the level of genetic prediction, theobromine and theophylline, which are metabolites of caffeine in the plasma, have the greatest direct or indirect impact on the overall thickness, surface area, and certain brain gyri of the cortex. This provides a clear direction for narrowing the scope of basic research and provides theoretical evidence for future MRI-based cohort studies on coffee intake, which are currently lacking. More importantly, this study provides a greater level of evidence for the current hotly debated claim that coffee intake may have long-lasting effects on brain cortex structure (46).

In recent years, epidemiological observations have consistently shown a time-dependent and dose-dependent impact of coffee consumption on changes in memory and reasoning abilities (48, 49). These effects were statistically significant even after adjusting for factors such as age, sex, education level, social behavior, diabetes status, hypertension, and coronary artery disease. The findings on hippocampal volume provide valuable insights, although proving the direct or indirect nature of these effects is challenging due to confounding factors in observational studies and potential biases in validity (such as residual confounding). At the level of coffee consumption and disease, a clear dose-dependent effect has been observed on certain diseases, particularly vascular dementia (50), type 2 diabetes (51), and Parkinson's disease (52), even after adjusting for BMI, lifestyle behaviors, and ethnicity. By combining the observed improvements with the impact of coffee on the brain cortex, our synthesized evidence provides a reference for potential new therapeutic pathways or targets for these diseases. Establishing causal relationships for these observational studies through long-term randomized controlled trials (the gold standard for causal hypotheses) based on MRI after prolonged coffee intake may be challenging due to ethical limitations and resistance to regular MRI reporting in the general population. Conducting such trials with sufficient sample sizes is also difficult and may not provide adequate evidence. In these circumstances, MR methods provide crucial information about causal relationships, which is of great value in guiding clinical research or basic research. These findings also indirectly support the findings of these observational studies, suggesting that coffee intake-related exposure factors have an impact on the brain cortex, and further speculation can be made that these effects, such as the observed improvement in memory in some RCTs with increased hippocampal volume, but the negative correlation between hippocampal volume changes and memory in the elderly population (53), can be partially explained by the dose-dependent effects of coffee intake on memory. The brain-gut axis is a bidirectional communication system between the brain and the digestive system (54) that interacts through neural, immune, and endocrine pathways. The role of this axis has been confirmed in various neurological and gastrointestinal diseases, such as depression and irritable bowel syndrome. In this study, evidence of overlapping high brain region responses confirmed the high overlap between the effects of coffee on the human cerebral cortex and the areas affected by diseases on the cerebral cortex. Coupled with existing evidence of the potential preventive effects of coffee on diseases, this discovery not only reveals a new perspective on the pathophysiology of diseases but also indicates the potential impact of coffee, a quantifiable impact of coffee on cerebral gyri and its interaction with diseases.

Our study has several notable strengths as well as several limitations. In terms of strengths, first, we introduced the MR design for the first time in this field, which reflects the causal association between coffee intake-related exposure and cortical and hippocampal structures. Additionally, as shown in Figure 1, our study population is predominantly from Europe, which greatly reduces population heterogeneity. Furthermore, careful inclusion of each cohort ensures a good overlap rate between the exposed and outcome populations, controlling for potential low-value causal estimates due to model overfitting. Second, the application of triangulation evidence is noteworthy. Both disease and coffee have been reported to potentially impact the human brain, and the overlapping effects may be related to the alleviation of diseases by coffee. We attempted to summarize the evidence in this area for the first time, providing precise evidence for subsequent basic experiments or clinical trials, including the overlap rate of affected brain regions with disease impact and the various affected brain regions in the Supplementary Table. In addition, we made careful selections within the triangulation evidence, such as the impact of coffee on diseases. Considering the possible reverse causal effects, we chose prospective cohort studies as much as possible to ensure logical sequencing. In terms of limitations, first, in the MR analysis, the choice of genetic instruments was limited in terms of the generalizability and applicability of causal effects worldwide, as our study population consisted mainly of Europeans. Second, although the analysis of the results demonstrated the impact of coffee-related exposure on cortical brain regions, the lack of GWAS data restricted comprehensive subgroup analyses, such as separate subgroup analyses based on sex, region, and age. Additionally, due to the scarcity of research, the impact of coffee on diseases as indicated by the triangulation of evidence has not been fully investigated in higher-tier studies such as randomized controlled trials. Finally, the limited number of SNPs involved in some analyses prevented us from conducting or fully conducting the three main MR methods, primarily IVW and sensitivity analyses. This may have led to potential biases that we were not aware of. In addition, statistical power values were not calculated in the supplementary analyses. Although power values have been used in other MR analyses in different fields and have demonstrated their usefulness, there are no available reference coefficients for the true correlation between the exposure and outcome variables. Thus, genetic predictions based on MR analysis can either amplify or attenuate effects. In the examination of the triangulation evidence, Pathway ① in Figure 3 was based on the assumption of possible true effects in the MR analysis. However, as mentioned earlier regarding the limitations of MR, this does not necessarily indicate the presence of true effects. In Pathway ②, we attempted to select prospective cohorts that reflected certain causal effects as evidence for this pathway. However, it is undeniable that some diseases, such as gait disorders, lack effective prospective cohorts, and the directionality may not be fully established. Therefore, Pathway ② for some of these diseases can only be considered a hypothesis based on existing cross-sectional analyses. For Pathway ③, clinical comparative studies effectively reflect the changes in cortical brain structures caused by diseases, and these regions had a high overlap with the regions affected by coffee. However, this does not completely indicate that the substances related to coffee intake and diseases act on the same pathway, although their effects on the outcome regions are consistent. Therefore, the conclusion that the pathways leading to changes in these overlapping regions are overlapping or concentrated in these regions needs to be interpreted cautiously, considering the observed improvement in diseases due to substances related to coffee intake in observational studies.

In conclusion, our study provides a platform for researchers to explore the factors related to coffee intake and their impact on brain structure. It has yielded some remarkable findings in terms of causal logic, such as the effects of theobromine and theophylline on the cortical level. This study also offers valuable directions for future clinical and basic research based on triangulation evidence. Future studies can utilize the identified regions to infer pathways or mechanisms. Additionally, more clinical studies with strong causal effects and triangulation evidence should be conducted to further explore the potential preventive applications for these diseases by investigating the effects of coffee on the brain.

## 5 Conclusions

This is the first comprehensive application of MR analysis and triangulation evidence revealing causal effects between coffee intake-related exposures and the cerebral cortex and hippocampus. Genetic prediction suggests that the increase in hippocampal volume and decrease in total cortical surface area from coffee intake are mainly driven by theobromine, a pathway involved in caffeine metabolism. This provides evidence for interpreting the effects of coffee intake and supports the presence of persistent changes in the brain due to coffee intake from a causal perspective. The overlap in the triangulation evidence revealed a significant correlation between the effects of gait disorders, major depression, Parkinson's disease, type 2 diabetes, suicide and the cerebral cortex, and the effects of coffee on the cerebral cortex. Further research is needed to investigate the specific mechanisms corresponding to these brain regions.

## Data availability statement

The original contributions presented in the study are included in the article/Supplementary material, further inquiries can be directed to the corresponding authors.

## Ethics statement

Ethical review and approval was not required for the study on human participants in accordance with the local legislation and institutional requirements. Written informed consent from the participants was not required to participate in this study in accordance with the national legislation and the institutional requirements.

## Author contributions

ZiL: Conceptualization, Data curation, Formal analysis, Investigation, Methodology, Resources, Software, Supervision, Validation, Visualization, Writing – original draft, Writing – review & editing. LX: Investigation, Validation, Writing – review & editing. XX: Investigation, Validation, Writing – review & editing. MS: Investigation, Validation, Writing – review & editing. YM: Investigation, Validation, Visualization, Writing – review & editing. HC: Investigation, Validation, Writing – review & editing. ZheL: Investigation, Resources, Software, Supervision, Validation, Writing – review & editing. ZhiL: Validation, Writing – review & editing, Software. JW: Validation, Writing – review & editing, Conceptualization, Formal analysis, Methodology, Supervision. YL: Conceptualization, Data curation, Formal analysis, Funding acquisition, Methodology, Project administration, Resources, Supervision, Writing – review & editing.
