# Supplementary material for: The relationship between coffee-related factors and cortical and hippocampal structure: a triangulation of evidence approach and Mendelian randomization research
Source: Front Nutr. 2024 May 21;11:1351067. doi: 10.3389/fnut.2024.1351067 (PMC11148385; doi:10.3389/fnut.2024.1351067)
Supplement: Supplementary file 1 [file Image_1.pdf]

Supplementary Information

The Relationship between Coffee-Related Factors and Cortical and Hippocampal Structure: A Triangulation of Evidence Approach and Mendelian Randomization Research

Figure 1. The results were significant in MR, and the sensitivity analysis of the overall main outcomes in a sufficient number of SNP exposures is shown as follows: (a) scatter plot of the effect of a 50% increase in genetic prediction of coffee intake on average thickness; (b) leave-one-out plot of the effect of a 50% increase in genetic prediction of coffee intake on average thickness; (c) funnel plot of the effect of a 50% increase in genetic prediction of coffee intake on average thickness.

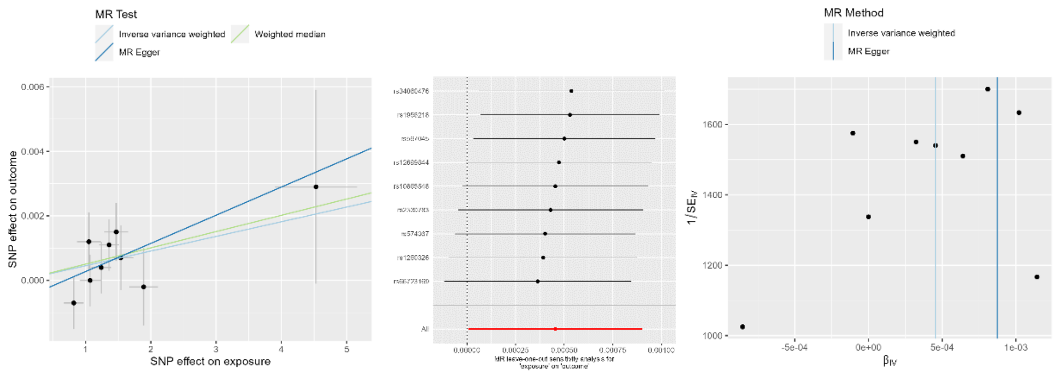

a b c

Figure 2. Significant MR analysis results for all brain regions (IVW analysis results only exist when the number of SNPs is greater than 1, and MR-Egger and weighted median analysis results only exist when the number of IVW is greater than 2).

Analysis items	Factors	Detailed	nSNP	beta(95%CI)
rostralmiddlefrontal with global weighted	Coffee	intake	9	1.797(0.017 to 3.576)
insula with global weighted	Caffeine	Plasma(metabolic pathway)	2	-15.463(-30.509 to -0.417)
parahippocampal with global weighted	Caffeine	Plasma(metabolic pathway)	2	-8.118(-14.939 to -1.297)
parsopercularis with global weighted	Caffeine	Plasma(metabolic pathway)	2	-16.283(-31.829 to -0.738)
supramarginal with global weighted	Caffeine	Plasma(metabolic pathway)	2	-39.953(-68.836 to -11.071)
temporalpole with global weighted	Caffeine	Plasma(metabolic pathway)	2	-4.776(-8.992 to -0.559)
parahippocampal without global weighted	Caffeine	Plasma(metabolic pathway)	2	-12.673(-21.075 to -4.271)
temporalpole without global weighted	Caffeine	Plasma(metabolic pathway)	2	-5.939(-10.572 to -1.306)
rostralmiddlefrontal with global weighted	Paraxanthine	Plasma(metabolic pathway)	2	43.559(0.014 to 87.104)
caudalanteriorcingulate without global weighted	Paraxanthine	Plasma(metabolic pathway)	2	17.708(4.369 to 31.046)
entorhinal without global weighted	Paraxanthine	Plasma(metabolic pathway)	2	8.466(0.84 to 16.093)
postcentral without global weighted	Paraxanthine	Plasma(metabolic pathway)	2	53.029(3.925 to 102.134)
rostralmiddlefrontal without global weighted	Paraxanthine	Plasma(metabolic pathway)	2	96.79(21.207 to 172.372)
caudalanteriorcingulate with global weighted	Theophylline	Plasma(metabolic pathway)	1	15.792(1.289 to 30.295)
lateralorbitofrontal with global weighted	Theophylline	Plasma(metabolic pathway)	1	30.629(4.116 to 57.142)
parsopercularis with global weighted	Theophylline	Plasma(metabolic pathway)	1	-26.824(-52.231 to -1.416)
superiortemporal with global weighted	Theophylline	Plasma(metabolic pathway)	1	-40.181(-74.438 to -5.923)
supramarginal with global weighted	Theophylline	Plasma(metabolic pathway)	1	-49.973(-97.443 to -2.504)
transversetemporal with global weighted	Theophylline	Plasma(metabolic pathway)	1	-10.318(-17.715 to -2.921)
fusiform without global weighted	Theophylline	Plasma(metabolic pathway)	1	-68.55(-121.841 to -15.26)
insula without global weighted	Theophylline	Plasma(metabolic pathway)	1	-40.053(-74.373 to -5.734)
isthmuscingulate without global weighted	Theophylline	Plasma(metabolic pathway)	1	-24.798(-46.634 to -2.961)
parsopercularis without global weighted	Theophylline	Plasma(metabolic pathway)	1	-45.091(-76.186 to -13.995)
precentral without global weighted	Theophylline	Plasma(metabolic pathway)	1	-78.759(-150.019 to -7.499)
superiortemporal without global weighted	Theophylline	Plasma(metabolic pathway)	1	-86.525(-143.954 to -29.096)
supramarginal without global weighted	Theophylline	Plasma(metabolic pathway)	1	-103.429(-173.819 to -33.039)
transversetemporal without global weighted	Theophylline	Plasma(metabolic pathway)	1	-16.072(-25.114 to -7.031)
posteriorcingulate with global weighted	Theobromine	Plasma	10	-7.374(-14.601 to -0.147)
parsopercularis with global weighted	Caffeine	Plasma	11	-17.314(-27.769 to -6.859)
inferiorparietal with global weighted	Paraxanthine	Plasma	16	-26.314(-47.321 to -5.307)
rostralmiddlefrontal with global weighted	Paraxanthine	Plasma	16	23.906(2.22 to 45.591)
superiorfrontal with global weighted	Paraxanthine	Plasma	16	25.457(5.689 to 45.225)
frontalpole without global weighted	Paraxanthine	Plasma	16	-1.683(-3.293 to -0.072)
inferiorparietal without global weighted	Paraxanthine	Plasma	16	-41.792(-72.103 to -11.481)
parsopercularis without global weighted	Paraxanthine	Plasma	16	-11.821(-22.378 to -1.264)
superiortemporal without global weighted	Paraxanthine	Plasma	16	-23.686(-43.19 to -4.182)
postcentral without global weighted	Theophylline	Plasma	11	-29.457(-57.514 to -1.4)

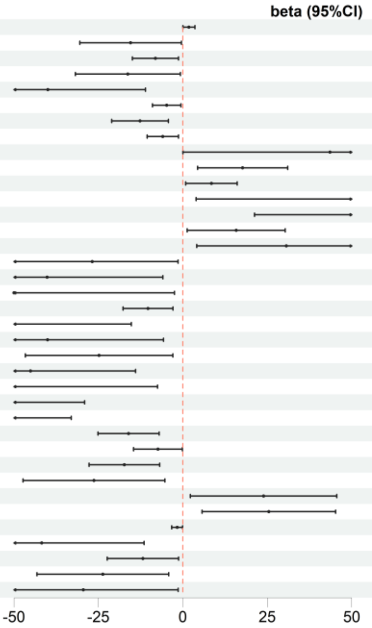

IVW (SA)

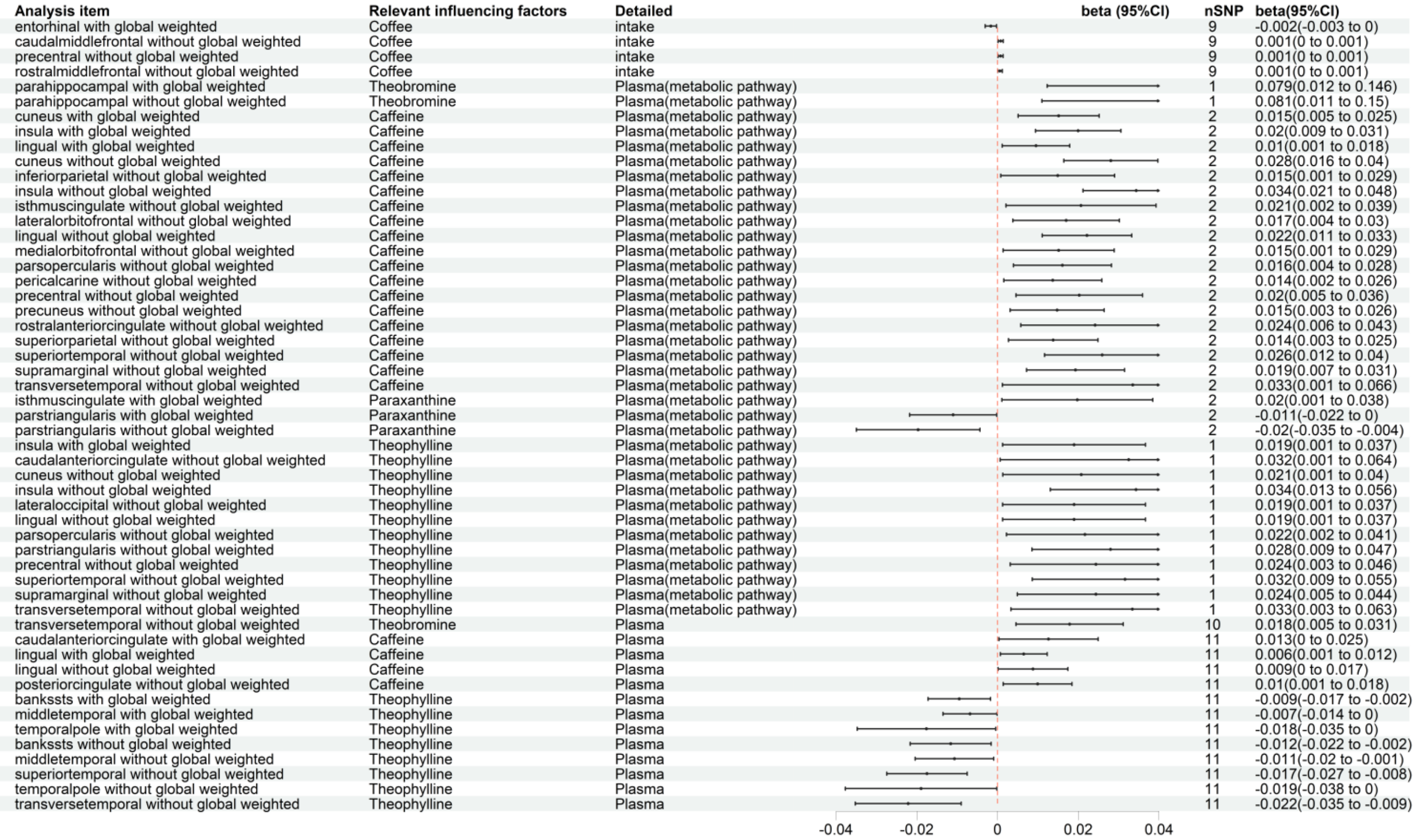

IVW (TH)

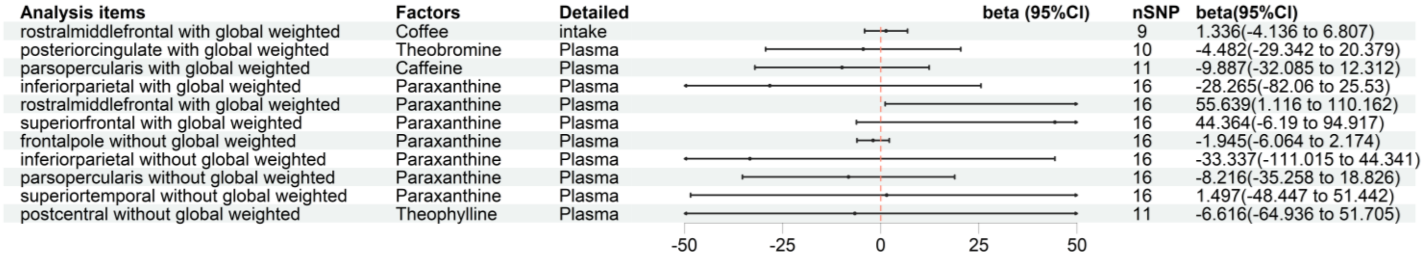

MR-Egger (SA)

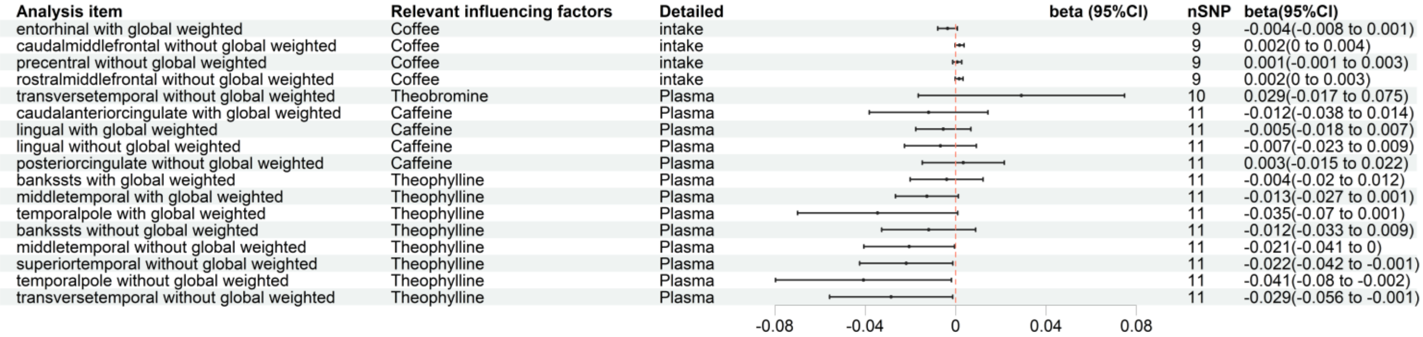

MR-Egger (TH)

Analysis items	Factors	Detailed	nSNP	beta(95%CI)
rostralmiddlefrontal with global weighted	Coffee	intake	9	2.373(0.095 to 4.65)
posteriorcingulate with global weighted	Theobromine	Plasma	10	-6.088(-15.732 to 3.556)
parsopercularis with global weighted	Caffeine	Plasma	11	-18.238(-31.725 to -4.751)
inferiorparietal with global weighted	Paraxanthine	Plasma	16	-23.566(-51.918 to 4.786)
rostralmiddlefrontal with global weighted	Paraxanthine	Plasma	16	26.553(-1.815 to 54.922)
superiorfrontal with global weighted	Paraxanthine	Plasma	16	18.467(-6.746 to 43.681)
frontalpole without global weighted	Paraxanthine	Plasma	16	-1.661(-3.699 to 0.376)
inferiorparietal without global weighted	Paraxanthine	Plasma	16	-28.586(-71.95 to 14.779)
parsopercularis without global weighted	Paraxanthine	Plasma	16	-9.052(-23.545 to 5.44)
superiortemporal without global weighted	Paraxanthine	Plasma	16	-27.855(-54.156 to -1.555)
postcentral without global weighted	Theophylline	Plasma	11	-23.011(-62.539 to 16.516)

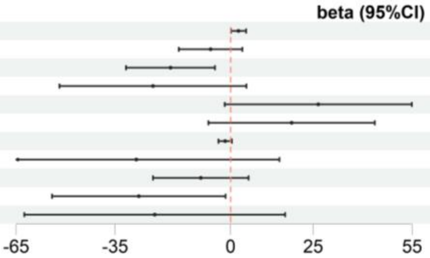

Weighted median (SA)

Analysis item	Relevant influencing factors	Detailed	nSNP	beta(95%CI)
entorhinal with global weighted	Coffee	intake	9	-0.002(-0.004 to 0)
caudalmiddlefrontal without global weighted	Coffee	intake	9	0.001(0 to 0.002)
precentral without global weighted	Coffee	intake	9	0.001(0 to 0.002)
rostralmiddlefrontal without global weighted	Coffee	intake	9	0.001(0 to 0.001)
transversetemporal without global weighted	Theobromine	Plasma	10	0.016(-0.003 to 0.034)
caudalanteriorcingulate with global weighted	Caffeine	Plasma	11	0.012(-0.004 to 0.028)
lingual with global weighted	Caffeine	Plasma	11	0.005(-0.003 to 0.014)
lingual without global weighted	Caffeine	Plasma	11	0.011(0 to 0.021)
posteriorcingulate without global weighted	Caffeine	Plasma	11	0.009(-0.003 to 0.021)
bankssts with global weighted	Theophylline	Plasma	11	-0.01(-0.021 to 0)
middletemporal with global weighted	Theophylline	Plasma	11	-0.006(-0.015 to 0.002)
temporalpole with global weighted	Theophylline	Plasma	11	-0.025(-0.049 to -0.002)
bankssts without global weighted	Theophylline	Plasma	11	-0.015(-0.029 to -0.001)
middletemporal without global weighted	Theophylline	Plasma	11	-0.011(-0.025 to 0.002)
superiortemporal without global weighted	Theophylline	Plasma	11	-0.014(-0.028 to 0)
temporalpole without global weighted	Theophylline	Plasma	11	-0.025(-0.05 to 0.001)
transversetemporal without global weighted	Theophylline	Plasma	11	-0.02(-0.038 to -0.003)

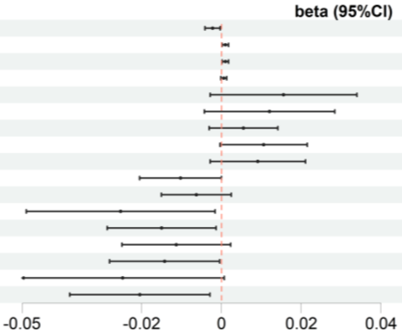

Weighted media (TH)

Figure 3. The results were significant in MR, and the sensitivity analysis of outcomes in a sufficient number of SNP exposures towards all brain regions is shown as follows: (a) scatter plot; (b) leave-one-out plot; (c) funnel plot.

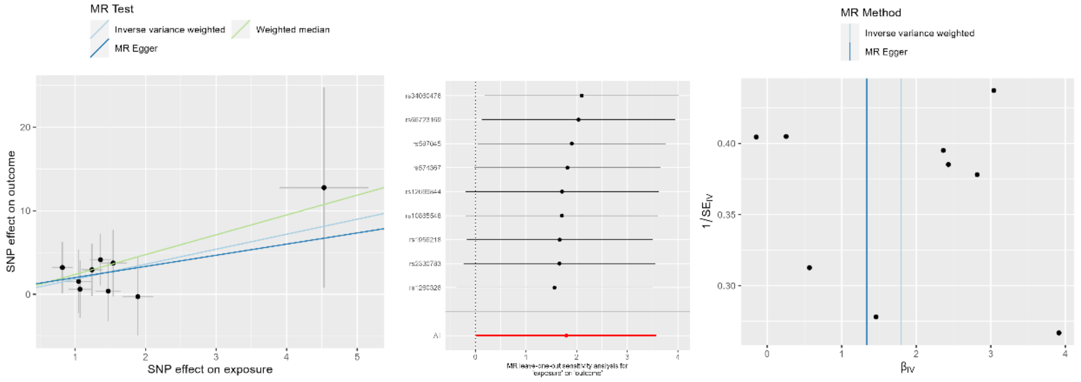

a b c

50% increase in coffee intake associated with rostralmiddlefrontal surface area (With global weighted)

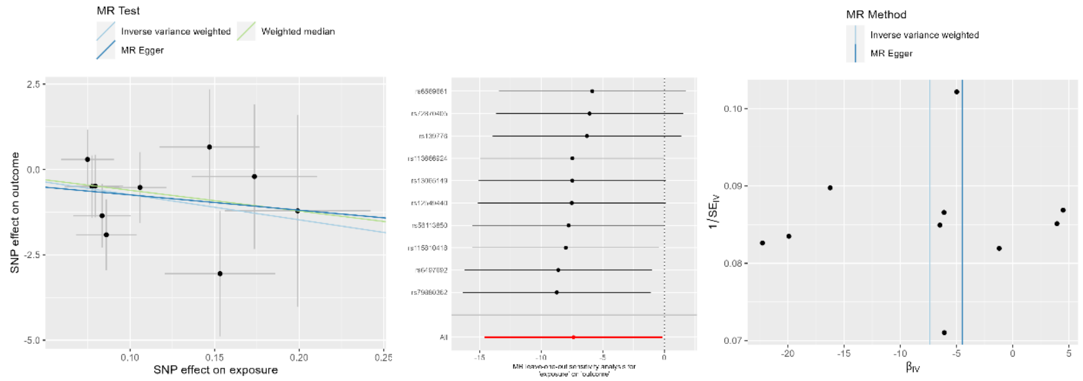

a **b** **c**

Plasma theobromine associated with posteriorcingulate surface area (With global weighted)

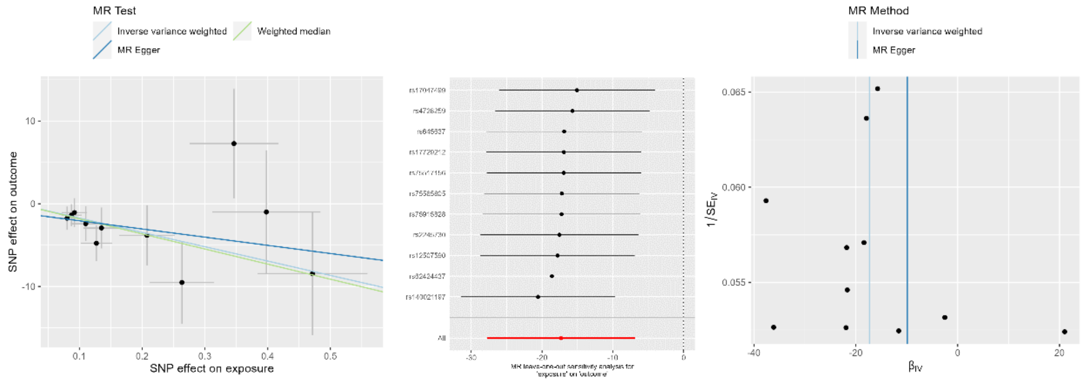

a **b** **c**

Plasma caffeine associated with parsopercularis surface area (With global weighted)

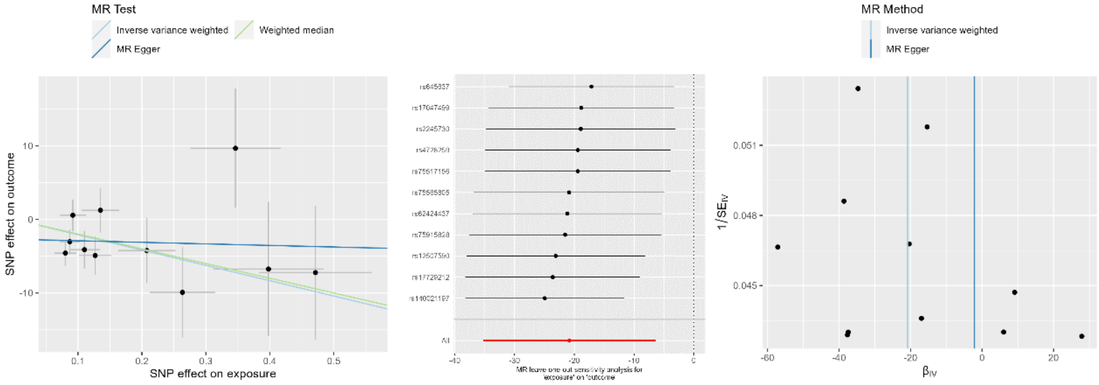

a **b** **c**

Plasma caffeine associated with parsopercularis surface area (Without global weighted)

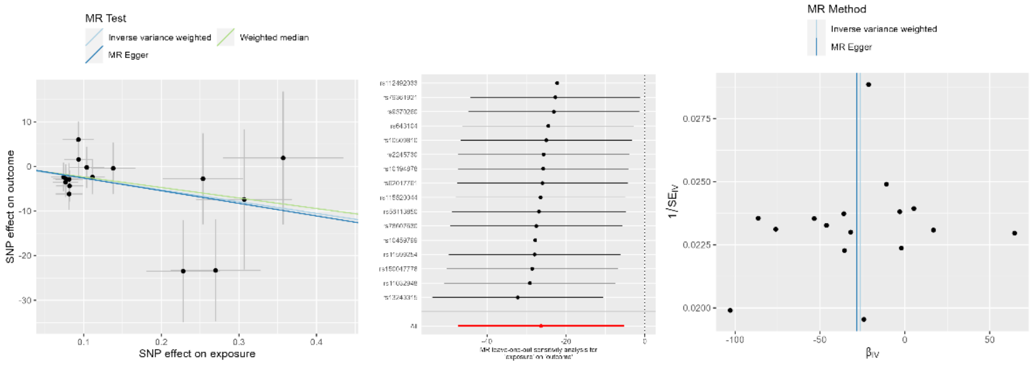

a **b** **c**

Plasma paraxanthine associated with inferiorparietal surface area (With global weighted)

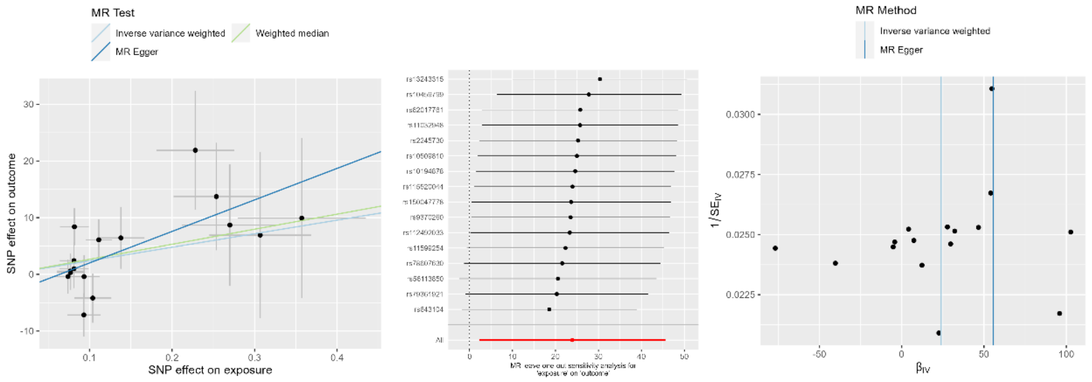

a **b** **c**

Plasma paraxanthine associated with rostralmiddlefrontal surface area (With global weighted)

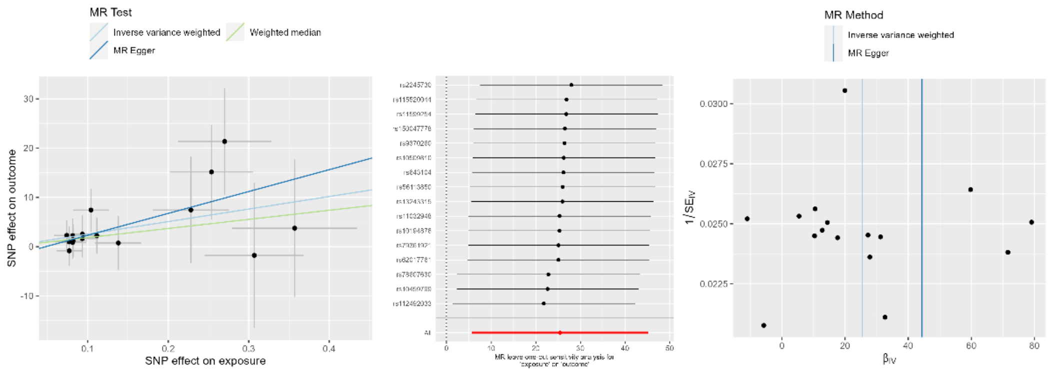

a **b** **c**

Plasma paraxanthine associated with superiorfrontal surface area (With global weighted)

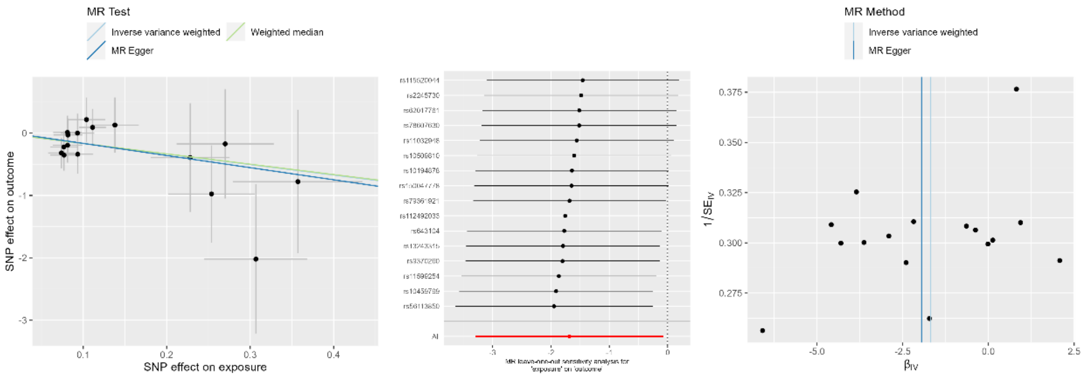

a **b** **c**

Plasma paraxanthine associated with frontalpole surface area (Without global weighted)

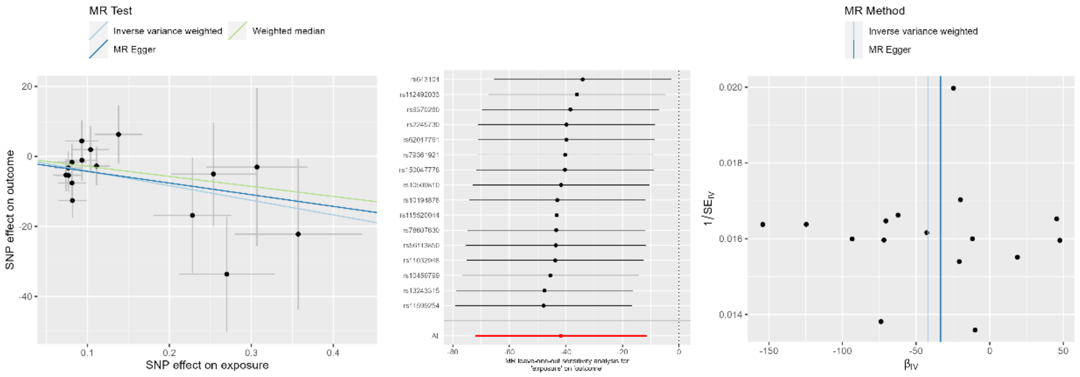

a **b** **c**

Plasma paraxanthine associated with inferiorparietal surface area (Without global weighted)

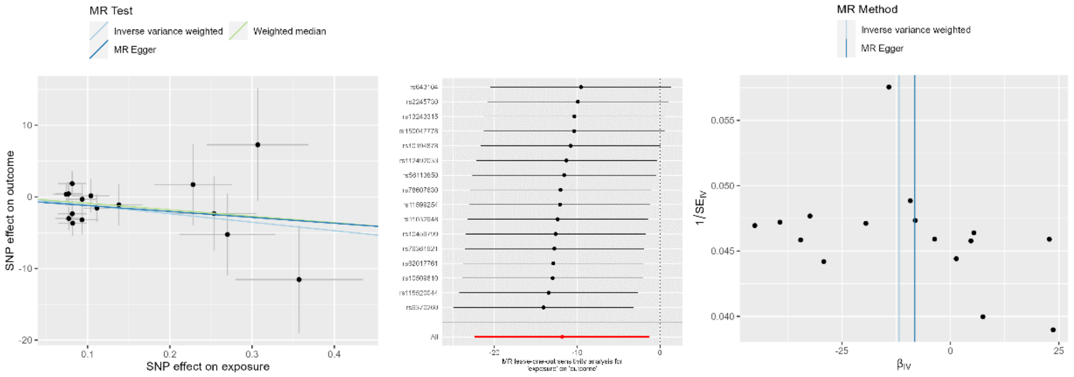

a **b** **c**

Plasma paraxanthine associated with parsopercularis surface area (Without global weighted)

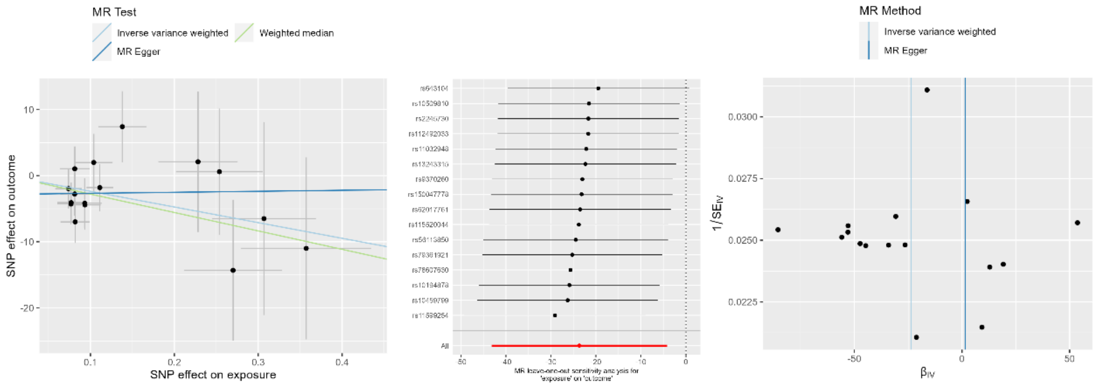

a **b** **c**

Plasma paraxanthine associated with superiortemporal surface area (Without global weighted)

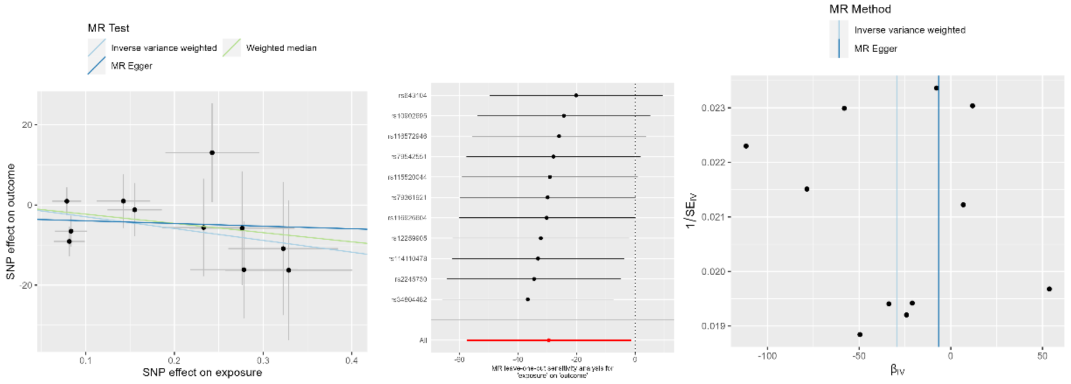

a **b** **c**

Plasma theophylline associated with postcentral surface area (Without global weighted)

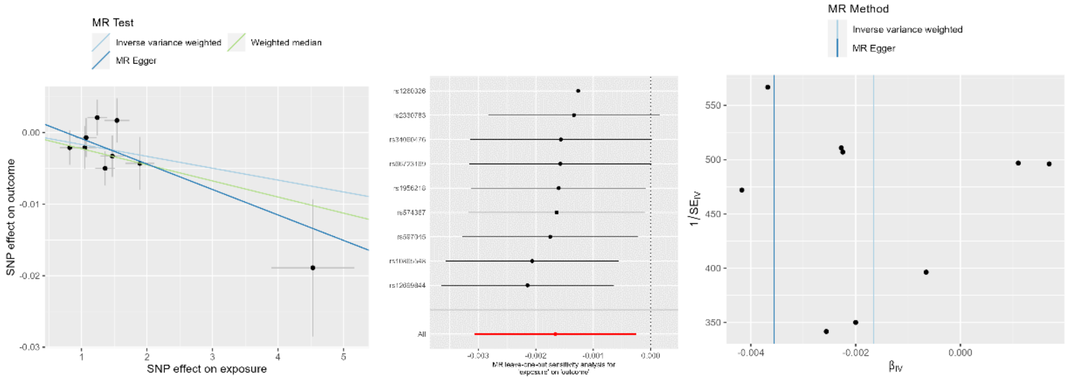

a **b** **c**

50% increase in coffee intake associated with entorhinal thickness (With global weighted)

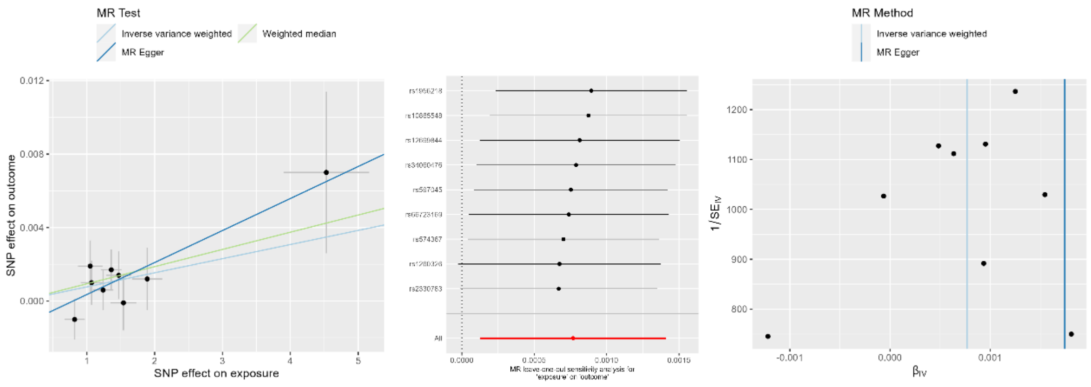

a **b** **c**

50% increase in coffee intake associated with caudalmiddlefrontal thickness (Without global weighted)

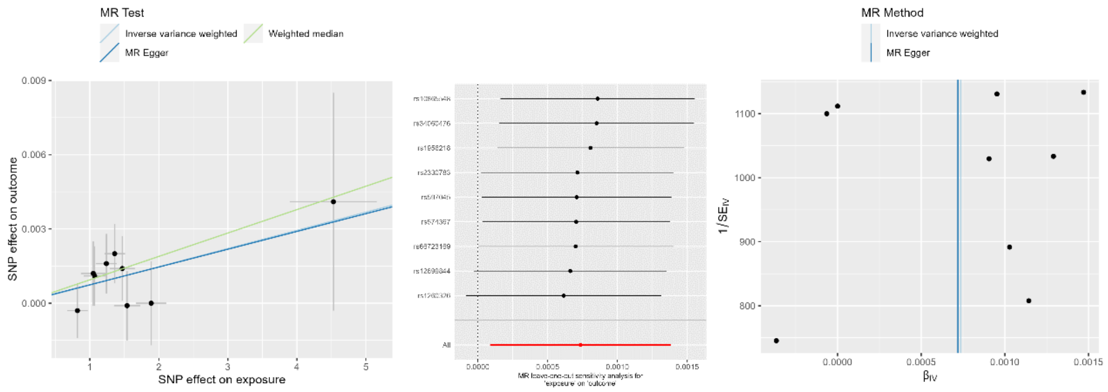

a **b** **c**

50% increase in coffee intake associated with precentral thickness (Without global weighted)

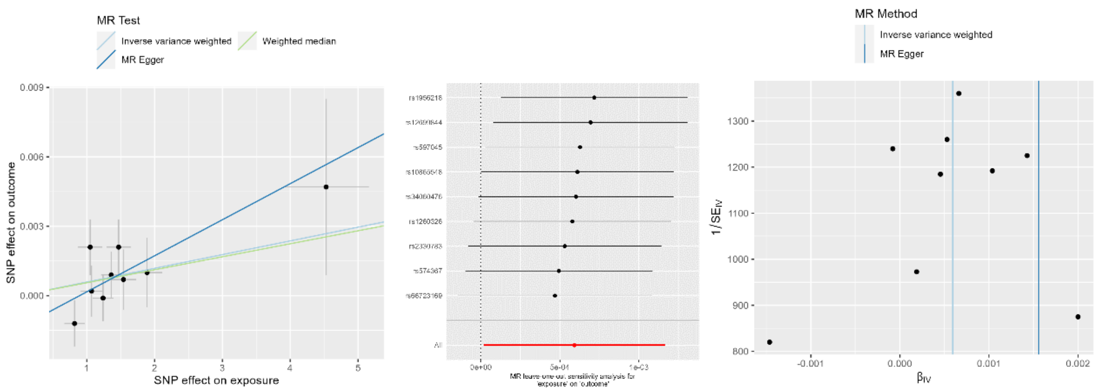

a **b** **c**

50% increase in coffee intake associated with rostralmiddlefrontal thickness (Without global weighted)

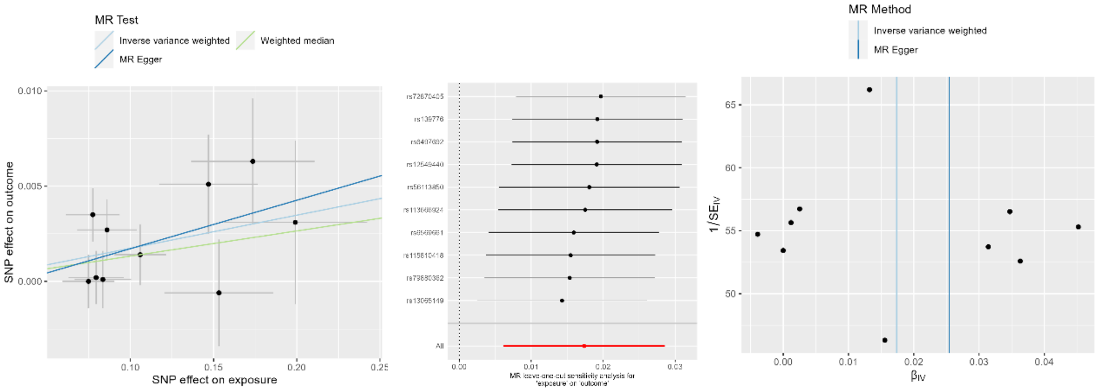

a **b** **c**

Plasma theobromine associated with transversetemporal thickness (With global weighted)

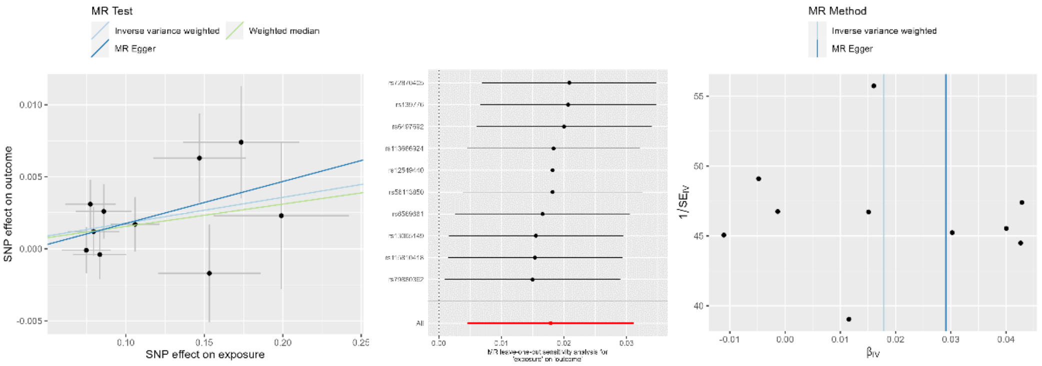

a **b** **c**

Plasma theobromine associated with transversetemporal thickness (Without global weighted)

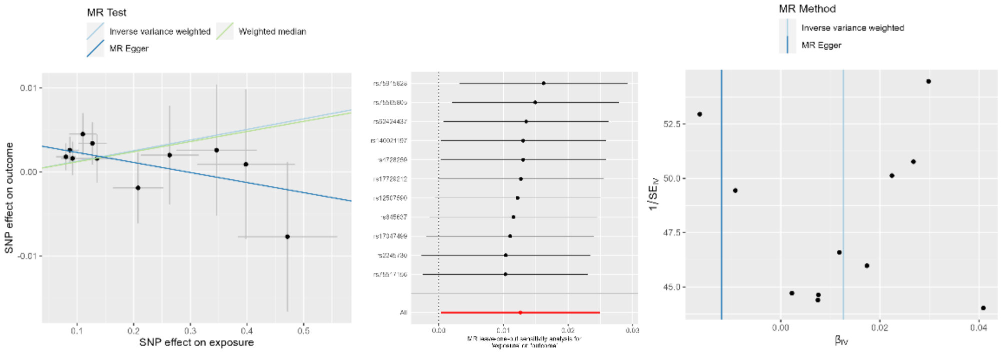

a **b** **c**

Plasma caffeine associated with caudalanteriorcingulate thickness (With global weighted)

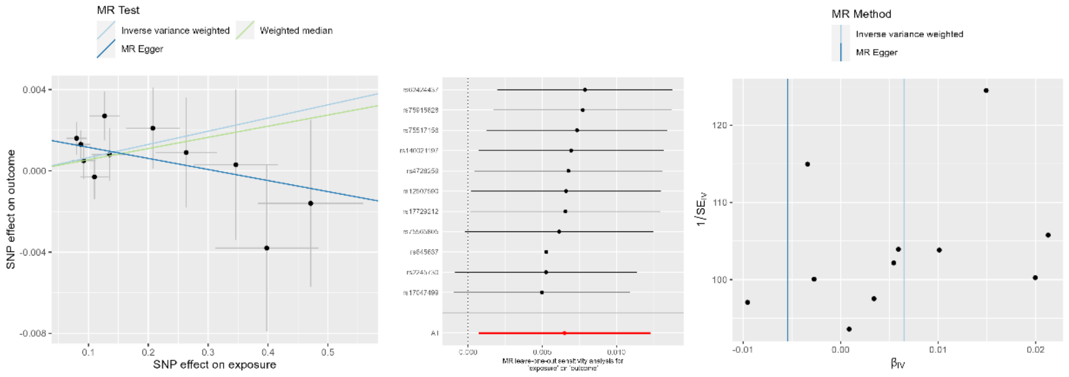

a **b** **c**

Plasma caffeine associated with lingual thickness (With global weighted)

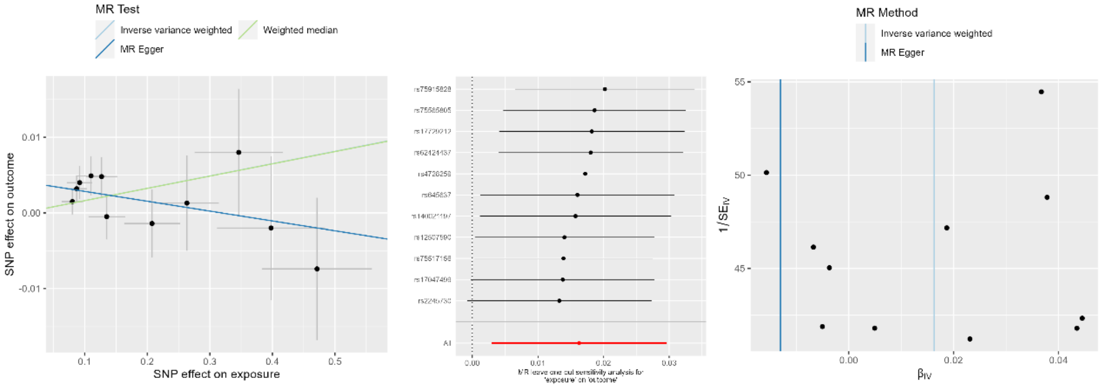

a **b** **c**

Plasma caffeine associated with caudalanteriorcingulate thickness (Without global weighted)

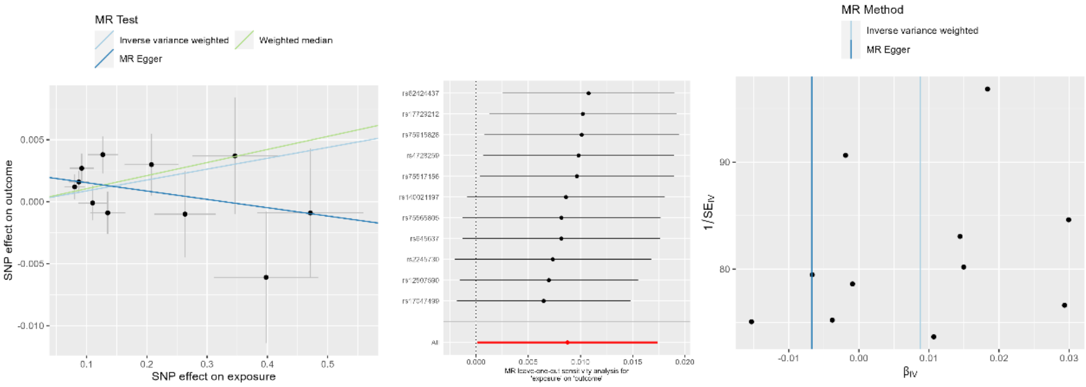

a **b** **c**

Plasma caffeine associated with lingual thickness (Without global weighted)

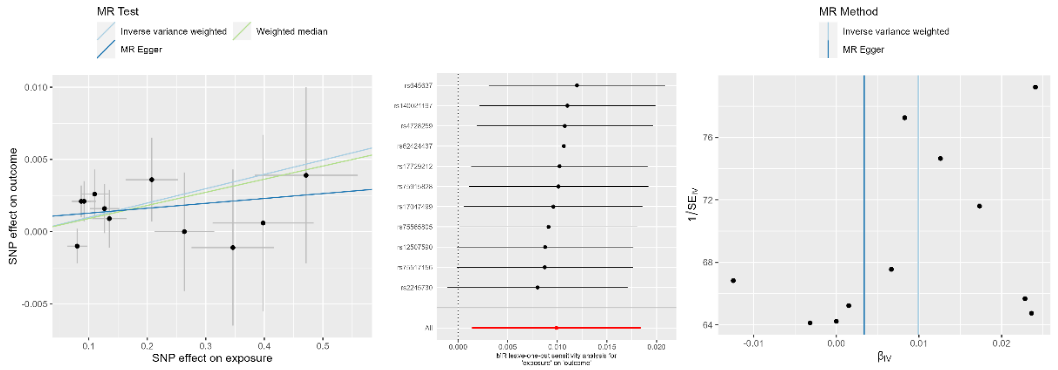

a **b** **c**

Plasma caffeine associated with posteriorcingulate thickness (Without global weighted)

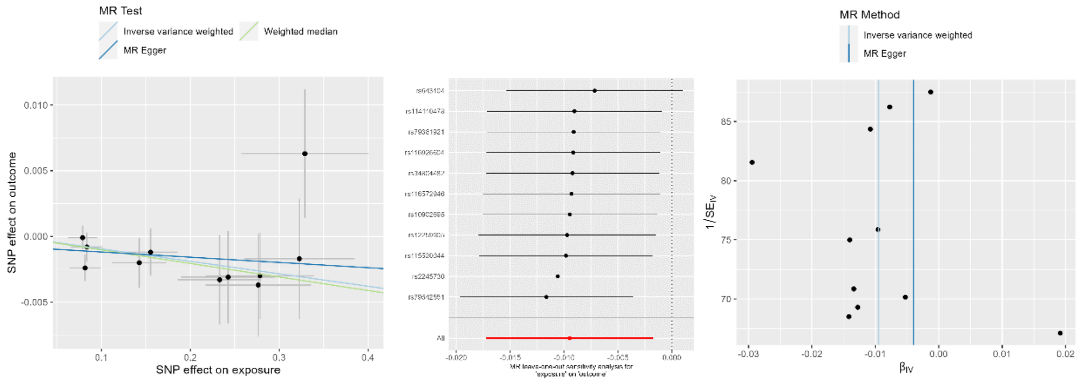

a **b** **c**

Plasma theophylline associated with bankssts thickness (With global weighted)

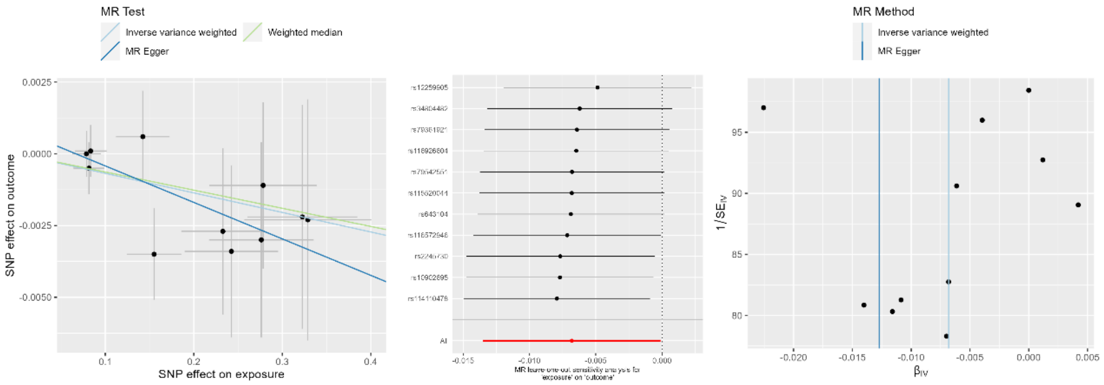

a **b** **c**

Plasma theophylline associated with middletemporal thickness (With global weighted)

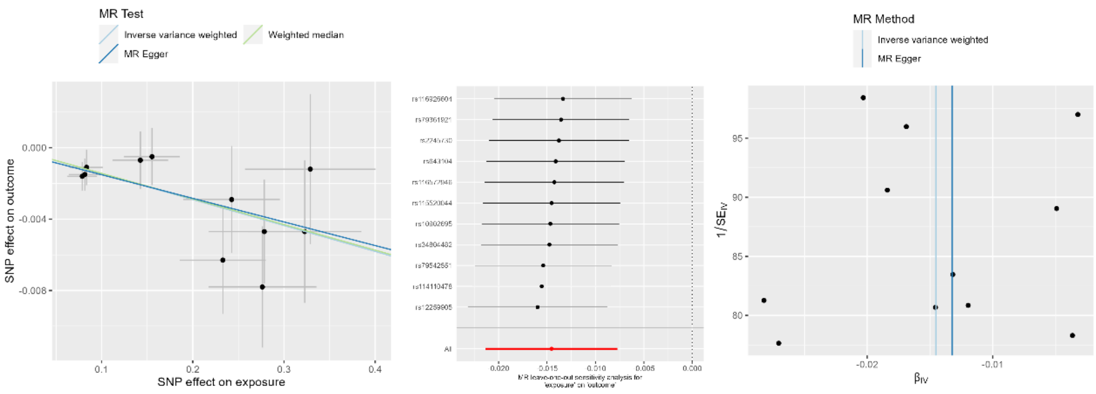

a **b** **c**

Plasma theophylline associated with superiortemporal thickness (With global weighted)

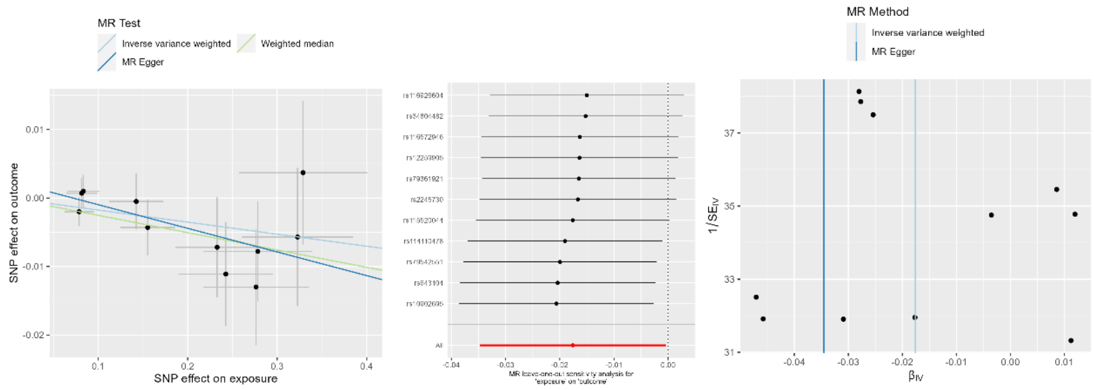

a **b** **c**

Plasma theophylline associated with temporalpole thickness (With global weighted)

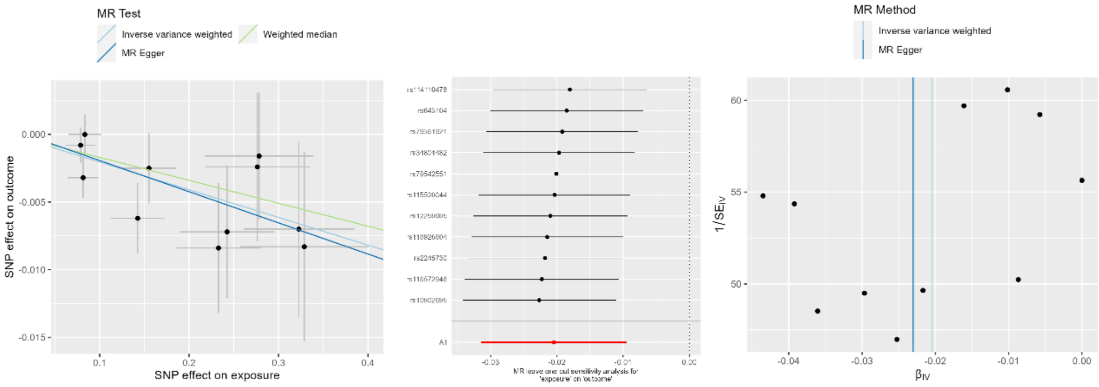

a **b** **c**

Plasma theophylline associated with transversetemporal thickness (With global weighted)

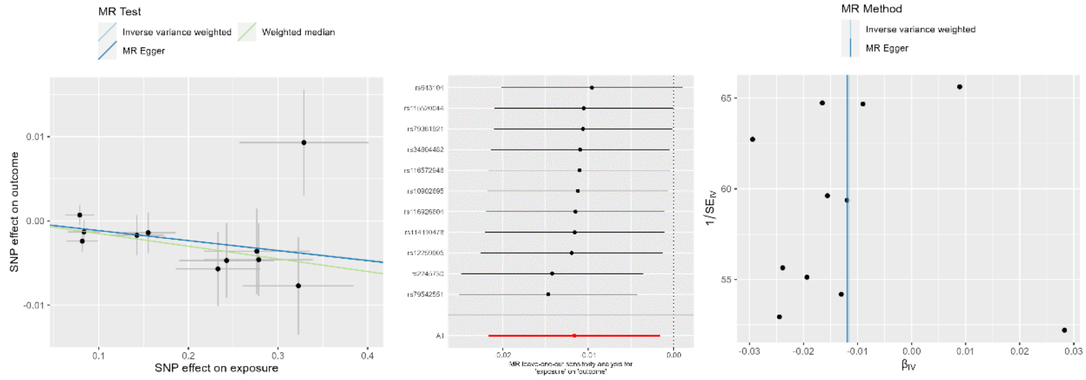

a b c

Plasma theophylline associated with bankssts thickness (Without global weighted)

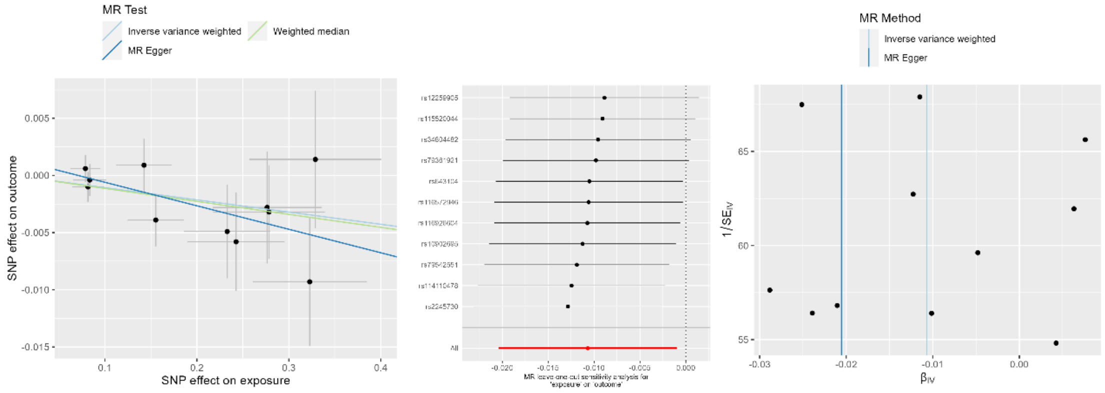

a b c

Plasma theophylline associated with middletemporal thickness (Without global weighted)

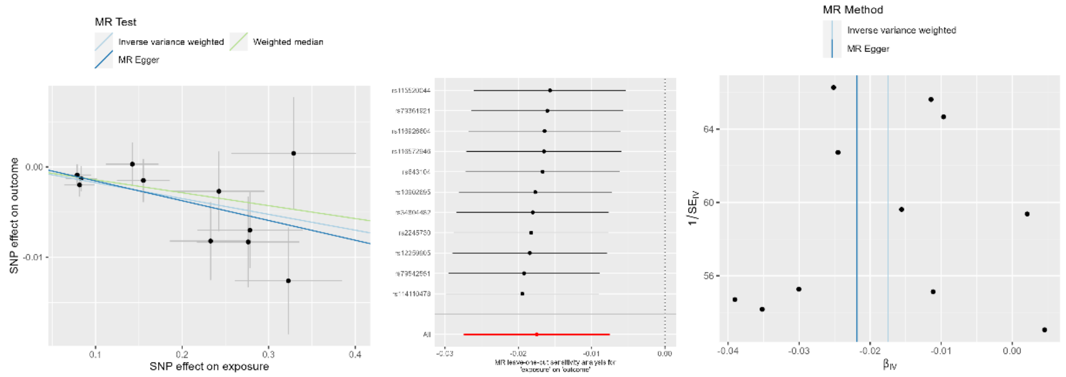

a b c

Plasma theophylline associated with superiortemporal thickness (Without global weighted)

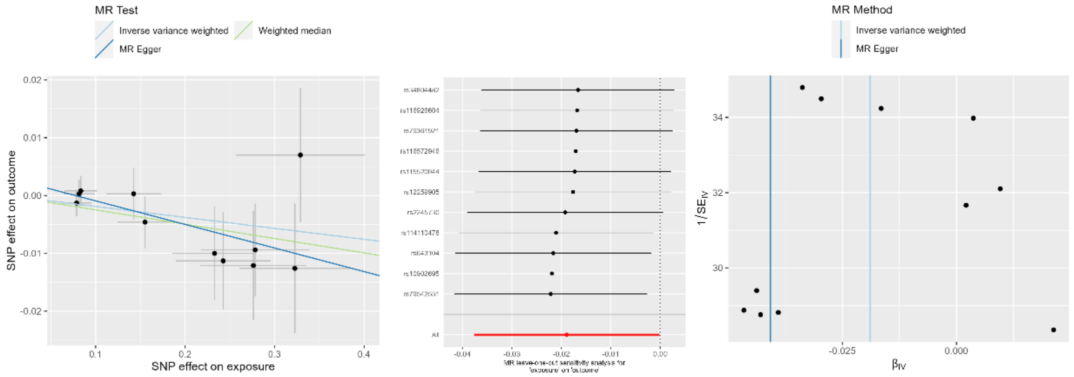

a **b** **c**

Plasma theophylline associated with temporalpole thickness (Without global weighted)

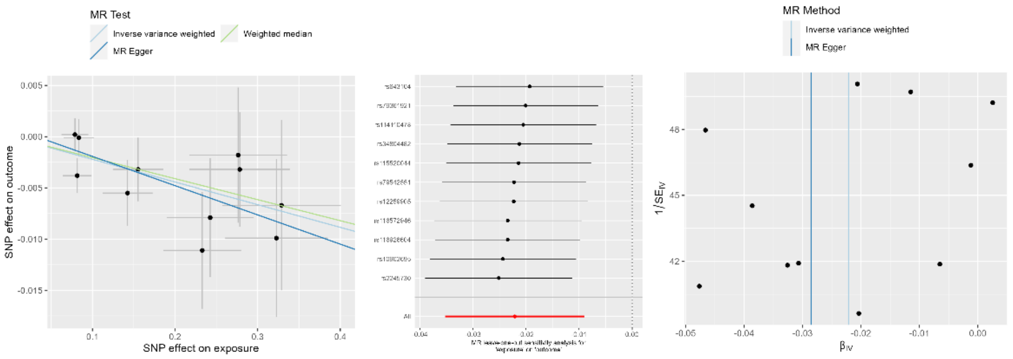

a **b** **c**

Plasma theophylline associated with transversetemporal thickness (Without global weighted)

Figure 4. The observational studies screening process for the impact of coffee on diseases is illustrated in the flowchart.

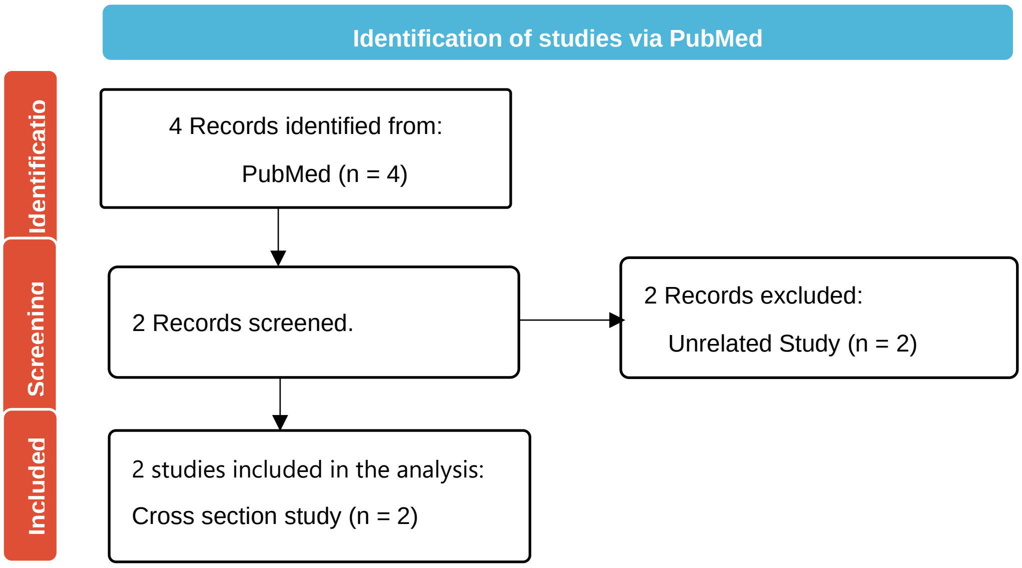

Coffee and Gait Disorders

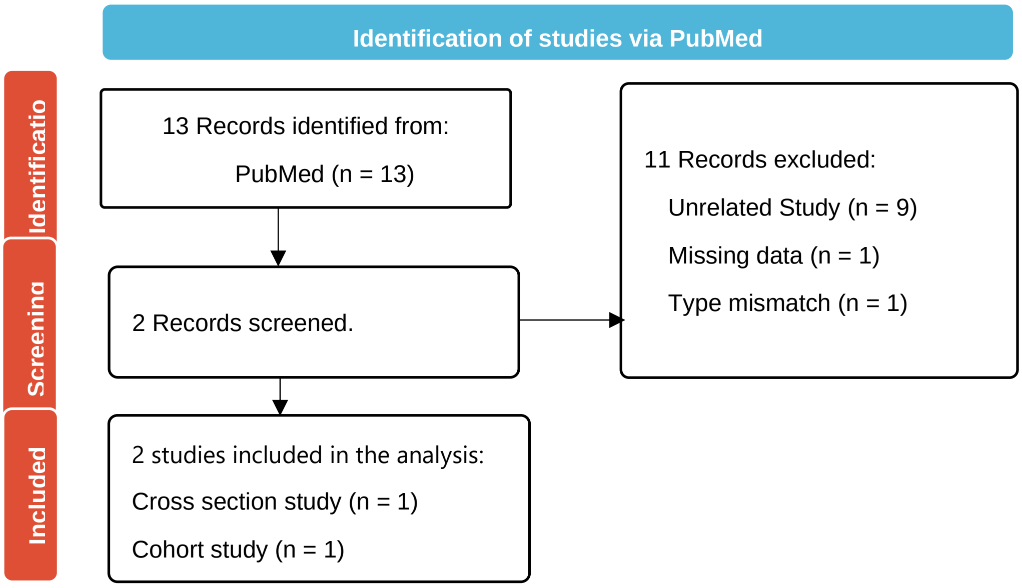

Coffee and Major depressive disorder

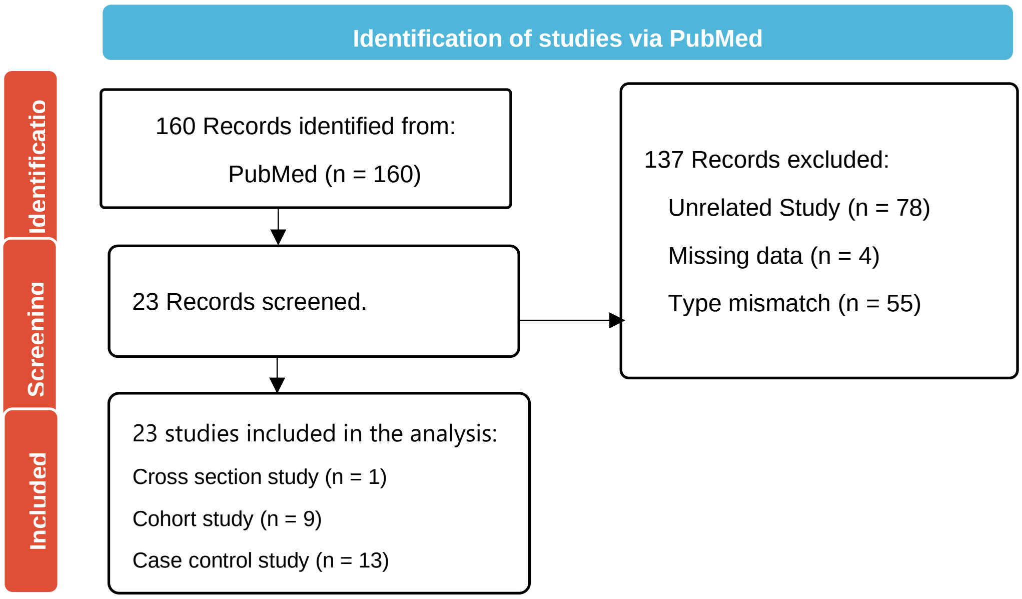

Coffee and Parkinson disease

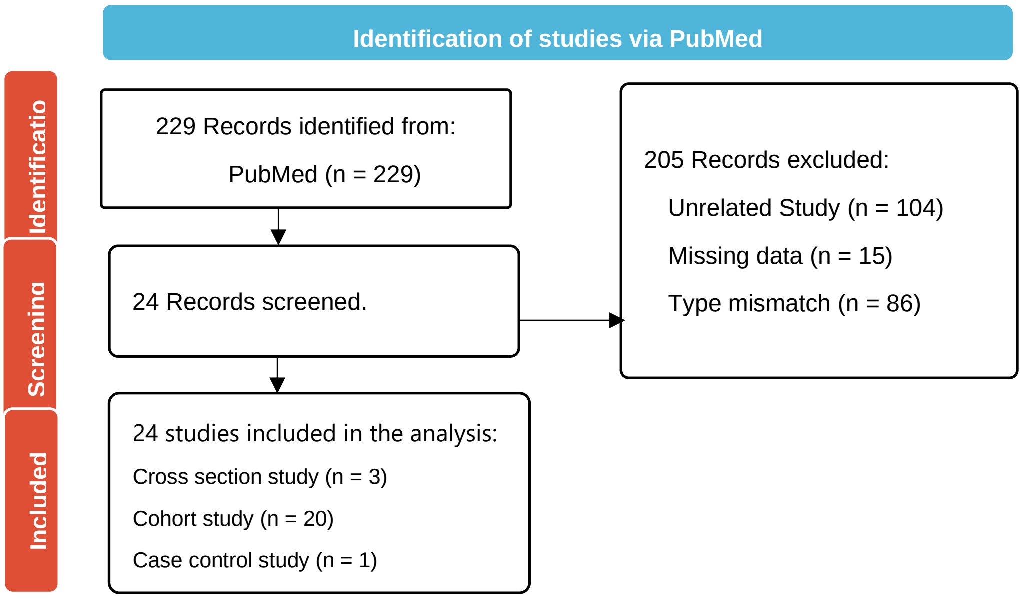

Coffee and Type 2 diabetes

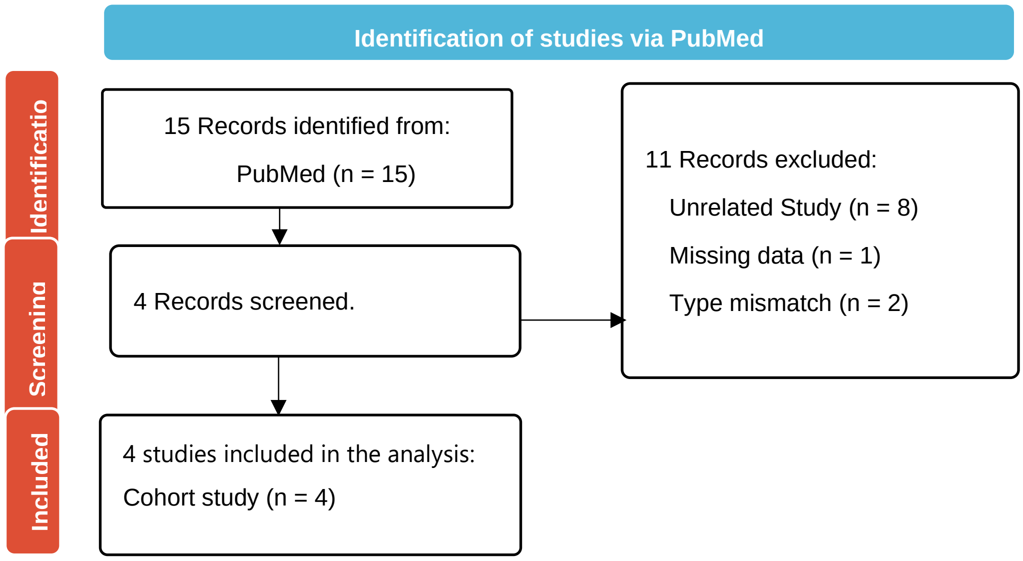

Coffee and Suicide
